# Deceased by default: Consent systems and organ-patient mortality

**DOI:** 10.1371/journal.pone.0247719

**Published:** 2021-03-17

**Authors:** Bart H. H. Golsteyn, Annelore M. C. Verhagen

**Affiliations:** Department of Economics, Maastricht University, Maastricht, The Netherlands; Harvard Medical School, UNITED STATES

## Abstract

Previous research shows that countries with opt-out consent systems for organ donation conduct significantly more deceased-donor organ transplantations than those with opt-in systems. This paper investigates whether the higher transplantation rates in opt-out systems translate into equally lower death rates among organ patients registered on a waiting list (i.e., organ-patient mortality rates). We show that the difference between consent systems regarding kidney- and liver-patient mortality rates is significantly smaller than the difference in deceased-donor transplantation rates. This is likely due to different incentives between the consent systems. We find empirical evidence that opt-out systems reduce incentives for living donations, which explains our findings for kidneys. The results imply that focusing on deceased-donor transplantation rates alone paints an incomplete picture of opt-out systems’ benefits, and that there are important differences between organs in this respect.

## 1. Introduction

Organ failure is a widely occurring medical condition with lethal consequences if left untreated. The most important treatment for end-stage organ failure is the transplantation of a donor organ. However, due to a shortage of donor organs, many patients die while waiting for an organ transplant (see, e.g., [1, 2]). Decreasing the mortality rate among organ patients is one of the primary goals of the organ donation policy agenda.

Almost every country has a system to formalize the default consent for deceased-donor organ donation. Systems in which the default is to become a donor after death are called “opt-out” or “presumed consent” systems. Systems in which the default is not to become a donor after death are “opt-in” or “informed consent” systems. Earlier research has shown that the number of transplantations from deceased donors is substantially higher in countries with opt-out systems than those with opt-in systems [3–8]. A positive correlation between opt-out systems and organ donation rates has also been found using hypothetical questions in laboratory experiments [9–11] and using survey questions on the willingness to donate an organ [12]. These findings seem to imply that opt-out systems reduce mortality among organ patients (see, e.g., [7]). However, the relationship between consent systems and organ-patient mortality has not yet been explicitly investigated yet. This paper aims to fill that gap in the literature.

The relationship between consent systems for organ donation and organ-patient mortality rates is not as obvious as it may seem, because a substantial difference in deceased-donor transplantation rates between consent systems also changes incentives. For example, a larger supply of organs from deceased donors reduces individuals’ incentives to become a living donor (see, e.g. [4, 13, 14]), or to purchase an organ or to obtain it abroad (i.e., organ trafficking, organ tourism, or legal organ trade within a transplant network). Consent systems may also change the incentives for medical professionals to enter, keep, or remove certain organ patients on/from the waiting list, and change governments’ and researchers’ incentives to invest in research and technology to increase organ-patient longevity. These incentives may affect behavior, and, as a result, lead to differences in organ-patient mortality between consent systems that are smaller to what one would expect based on the differences in deceased-donor transplantation rates alone.

This paper is the first to analyze whether organ-patient mortality rates are indeed lower in countries with opt-out consent systems for organ donation compared to those with opt-in systems. We study between-country differences, using data from newsletters from the Organización Nacional de Trasplantes on the number of kidney, liver, heart, and lung transplantations performed and the number of patients who died while registered on a waiting list to receive a donation of one of those organs. We gathered data from several other sources to determine whether a country has an opt-out or opt-in system. Combining these data, we create the largest data set available on organ-patient mortality and organ transplantations; our sample consists of 69 countries across a period of 15 years (2001–2015, unbalanced panel). Additionally, we obtained data on several potentially confounding factors from the World Bank, the World Health Organization, and the World Values Survey.

There is a considerable difference between a patient with kidney disease (who may remain on dialysis for many years or may receive a live donor kidney), a patient with heart disease (who can receive a left ventricular assist device or “artificial heart” as a bridge to the transplant), a patient with lung disease (who may choose a ventilator), and a patient with liver disease (for whom live split-liver donation is possible, but artificial devices are not available). We therefore show separate analyses for each organ and do not pool the organs. We estimate two regressions for kidneys, livers, hearts, and lungs separately, while controlling for year fixed-effects as well as for a country’s GDP, general mortality rates, health expenditures, and religious denomination. The first regression shows the relationship between countries’ consent systems for organ donation and the number of deceased-donor transplantations per million population (hereafter, “deceased-donor transplantation rates”). The second regression shows the relationship between consent systems and patients who died while on a waiting list for a donor organ per million population (hereafter, “organ-patient mortality rates”). By estimating the two equations as seemingly unrelated regressions, we can analyze whether the correlation between opt-out systems and deceased-donor transplantation rates is significantly different from the correlation between opt-out systems and organ-patient mortality rates.

For hearts, we find that opt-out systems have a small but statistically significant advantage in terms of deceased-donor transplantation rates, and that this translates into similarly lower heart-patient mortality rates compared to opt-in systems. For lungs, however, deceased-donor transplantation rates do not differ significantly between consent systems; this is reflected in the mortality rates, which are also not significantly different from each other between consent systems. Although the advantages of opt-out systems are by far the largest for deceased-donor kidney and liver transplantation rates, these are also the organs for which the differences in organ-patient mortality rates are significantly smaller (i.e., closer to zero) than the differences in deceased-donor transplantation rates between consent systems.

We investigate which incentives could explain the surprisingly small differences between consent systems regarding kidney- and liver-patient mortality rates. For kidneys, we find empirical evidence that lower incentives to become a living donor in opt-out systems, reflected by lower rates of living donations, can explain our findings: the difference between consent systems in kidney-patient mortality rates is similar to what could be expected from the total kidney transplantation rates (i.e., from deceased and living kidney donors combined). For livers, differences in organ-patient mortality rates between consent systems remain significantly smaller than the differences in transplantation rates, even when transplantations from living donors are considered. We do not find empirical evidence that opt-out systems are related to different incentives for kidney and liver patients to purchase an organ on the black market (i.e., organ trafficking or organ tourism): excluding countries with reports of organ trade as either a donor or recipient country does not change our main results.

We investigate the robustness of our results in various sensitivity checks. One potential measurement issue is related to the earlier stated hypothesis that incentives to enter, keep, or remove patients from the waiting list may differ between consent systems. More lenient policies regarding waiting list entry not only affect the probability of patients dying from other factors than organ failure (e.g., co-morbid conditions); they also increase the number of patients registered on the waiting lists and thereby, by construction, the number of deaths on the waiting lists. However, the empirical evidence does not support these hypotheses: taking the conservative approach of assuming that if an organ patient was removed from the waiting list because (s)he was too sick to receive a transplant implies that the organ patient died, does not change our results. Our results also remain unchanged when we control for waiting list length, indicating that countries with different consent systems do not have substantially different policies regarding waiting list entry. These two robust results indicate that, although fatal co-morbid conditions affect the number of deaths on the waiting lists, it is unlikely that they substantially affect the difference in organ-patient mortality rates between consent systems. The results also remain robust when we reduce country heterogeneity by only including Europe, North America, Australia, and New Zealand, when we use different methods to deal with zero values (i.e., excluding zero values and estimating Tobit regressions), and when we deal with potential outliers by truncating the data at the 99^th^ percentile.

The findings provide an important contribution to the literature on organ donation and thereby provide new insights for the discussion on the advantages and disadvantages of the two consent systems for organ donation. First, they highlight that the advantages and disadvantages differ substantially by organ. This is an important addition to the current literature on the topic, which often focuses on one single organ (typically either kidneys or livers). Second, we find empirical evidence that opt-out systems decrease the incentives to become a living kidney donor. Although we are not the first to find this result (see, e.g. [4, 13, 14]), the fact that living kidney transplantation rates are on average lower in opt-out systems remains a (dis)advantage of opt-out systems that receives little to no attention in the debate on the pros and cons of different consent systems. Further research is needed to better understand the channels through which consent systems can affect organ-patient mortality rates, channels such as technological investments regarding organ-patient longevity and organ trade through transplant networks, as well as to what extent and why these channels differ by organ.

This paper proceeds as follows: Section 2 discusses the empirical strategy, Section 3 discusses the data, and Section 4 gives the results. Section 5 concludes.

## 2. Empirical strategy

### 2.1 The relationship between consent systems and organ-patient mortality rates

The aim of this paper is to analyze whether organ-patient mortality rates differ between countries with opt-out or opt-in consent systems for organ donation. In our main specifications, we estimate the following model for kidneys, livers, hearts, and lungs separately:

$$mortality_{ct}=\alpha_{0}+\alpha_{1}optout_{c}+\delta_{1t}+X\prime_{ct}\gamma +\varepsilon_{1ct}.$$ (1)

In this equation, *c* indexes the country, and *t* indexes time in years. For simplicity, organ-specific subscripts are suppressed from the equation. *Mortality* is the organ-patient mortality rate, i.e., the number of people who died throughout year *t* while on a waiting list to receive either a donor kidney, liver, heart, or lung, divided by the size of the population (in millions) of that person’s country of residence. The variable *optout* is an indicator variable with a value of 1 if a country has an opt-out consent system for organ donation and 0 if that country has an opt-in system. In our main specification, we exclude countries that changed their consent system within our data window (see more about this in Section 2.4). Therefore, the opt-out dummy variable does not vary over time. We also control for year fixed effects *δ* as well as for ***X’***; a vector of country characteristics, i.e., the country’s GDP, health expenditures, age-standardized death rates (all causes), and religious denomination (see Section 3 and Appendix 1 in S1 File for more information). All regressions also include dummy variables for missing values on the independent variables. ε is the normally distributed error term, which is allowed to correlate with the error-term of Eq (2) (more on this in Section 2.3 below). Because organ-patient mortality rates are likely to be correlated over time within countries, we cluster the standard errors at the country level.

Please note that for simplicity, we sometimes use the term “effect” to describe the correlation between consent systems and our dependent variables. However, this paper does not identify causal effects of consent systems.

### 2.2 The relationship between consent systems and deceased-donor transplantation rates

Besides the analyses on organ-patient mortality, we also analyze the relationship between consent systems and deceased-donor transplantation rates. If deceased-donor transplantation rates are the only mechanism through which consent systems affect organ-patient mortality rates, the relationship between consent systems and organ-patient mortality rates should be similar (although with the opposite sign) to the relationship between consent systems and deceased-donor transplantation rates. We estimate the following model for each organ separately (again, organ-specific subscripts are suppressed for simplicity):

$$DDTX_{ct}=\beta_{0}+\beta_{1}optout_{c}+\delta_{2t}+X\prime_{ct}\mu +\varepsilon_{2ct}.$$ (2)

In this equation, *DDTX*_*ct*_ stands for the deceased-donor transplantation rate in country *c* in year *t*, i.e., the number of transplantations from deceased donors divided by the size of the population (in millions). Based on previous findings in the literature, we expect ${\hat{\beta }}_{1}$ to be larger than zero, i.e., that deceased-donor transplantation rates are higher in countries with opt-out systems. To reduce unobserved heterogeneity and improve the efficiency of estimator ${\hat{\beta }}_{1}$, we again include year fixed effects *δ* as well as ***X’***, which is a vector of variables that are identical to those in Eq (1). Again, we cluster the standard errors at the country level.

### 2.3 Seemingly unrelated regressions

If the difference between consent systems regarding organ-patient mortality rates is significantly different from the difference between the systems regarding deceased-donor transplantation rates (i.e., if ${\hat{\alpha }}_{1}$+${\hat{\beta }}_{1}$≠0), consent systems must affect organ-patient mortality rates through channels other than deceased-donor transplantation rates alone. However, even if there would be no one-to-minus-one relationship between the opt-out effect on deceased-donor transplantation rates and the opt-out effect on organ-patient mortality rates, it is unrealistic to assume that these two effects are completely unrelated to each other. This means that it is unrealistic to assume that the covariance between the error terms of regressions (1) and (2) is equal to zero. By estimating both regressions simultaneously using a seemingly unrelated regression (SUR) technique, the covariance between both regressions can be taken into account when testing whether ${\hat{\alpha }}_{1}$+${\hat{\beta }}_{1}$ = 0. Estimating Eqs (1) and (2) as SUR rather than as separate regressions does not change the regression results in any way.

### 2.4 Using countries that changed consent systems

Although it is tempting to try to exploit the fact that some countries in our data changed their consent system for organ donation within our data window, this would be inappropriate for several reasons. First, a country’s decision to change its consent system is likely to be endogenous, i.e., related to previous trends in organ-patient mortality rates, the population’s attitudes toward organ donation, or technological advances in increasing organ-patient longevity, for instance. Therefore, estimates based on within-country analyses do not necessarily reveal causal effects of a consent system change but rather pick up the continuation of a previously existing trend.

Second, even if the decision to change the consent system was exogenous, implementing this change may have lagged effects on transplantation rates and therefore on organ-patient mortality rates: it may take several years before countries have sufficiently increased their health care capacity (e.g. medical equipment, hospital beds, and medical personnel) to use the entire additional supply of organ donors. Therefore, merely adjusting the opt-out dummy from the year of the consent system change onward, thereby ignoring any lagged effects, would likely lead to an estimation of *α*_1_ and *β*_1_ that is biased toward zero. However, trying to take into account the lagged effects poses problems as well because it is uncertain how big the lag would be and because the size of the lag may vary from one country/consent system change to another. Moreover, even if the size of the lag would be known, the fact that there is a lag makes it substantially less likely that nothing else changed that may affect transplantation or organ-patient mortality rates since the consent system changed, again leading to biased estimates of *α*_1_ and *β*_1_.

Finally, only a few countries in our sample switched from one consent system to another within our data window. Therefore, the statistical power to study within-country differences is limited, which may lead to biased estimates of *α*_1_ and *β*_1_.

## 3. Data

### 3.1 Data sources

We obtained the data from a great variety of sources, leading to the largest data set available to study the relationships between consent systems and organ-patient mortality rates and transplantation rates. In this section, we briefly introduce the data. In Appendix 1 and Appendix 2 in S1 File, we give the sources of the variables and discuss data selection.

Our main data source is newsletters from the Organización Nacional de Trasplantes. From Newsletter Transplant 2002 (reporting data on calendar year 2001) onward, the newsletters include data on the number of kidney, liver, heart, and lung transplantations performed, as well as the number of patients who died while on a waiting list to receive a donor kidney, liver, heart, or lung. For each organ, we divide both variables by the size of the population (in millions) to obtain the rates of organ-patient mortality and transplantations.

Between 2001 and 2015, the number of countries included in our sample increased from 35 to 66. In Table B in the S1 File, we show which countries are included in the newsletters by year. In additional analyses, we also use data from the Newsletters Transplant on the annual number of kidney and liver transplantations from living donors, the annual number of patients who were on the waiting list to receive an organ on December 31 in each year, and the annual number of patients who entered the waiting list for the first time in that year.

From other sources, we obtained information on whether countries have opt-in or opt-out consent systems (see Table C in the S1 File). Every year, around 60% of the countries in the sample had an opt-out consent system for organ donation. We control for countries’ annual gross domestic product (GDP) per capita, annual health expenditures per capita, annual general mortality rates (all causes and age standardized), and religion (measured as the average share of the population between 2000 and 2014 that indicated they were Roman Catholic). We obtained these data from the World Bank, the global health expenditures database of the WHO, the WHO mortality database, and the World Values Survey, respectively (see Appendix 1 in S1 File for more information). For one of the robustness checks, we also obtained data from Eurotransplant and Scandiatransplant on the number of patients who were removed from organ transplantation wait lists.

### 3.2 Descriptive statistics

Most deceased-donor transplantations (per million population: pmp) are kidney transplantations, and most patients who died while on the waiting list (pmp) were waiting for a kidney transplant (see Fig 1 in the S1 File). This indicates that the organ shortages problem is driven by a kidney shortage. More than half of all deceased-donor transplants (pmp) are kidney transplants (55%), 26% are liver transplants, 9% are heart transplants, and 10% are lung transplants. Of all patients who died while on a waiting list for organ transplantation (pmp), 53% were on a waiting list for kidney transplants, 25% were on the liver waiting list, 12% were on the heart waiting list, and 10% were on the waiting list for a lung transplant.

In 2015, the average deceased-donor transplantation rate in countries with an opt-out consent system was 22.2 pmp for kidneys (17.6 in opt-in systems), 9.3 (7.0) for liver transplantations, 3.6 (2.9) for heart transplantations, and 2.9 (3.9) for lung transplantations. Although there are minor fluctuations and differences between consent systems, every year the organ-patient mortality rate is around 5 pmp among patients who were on a kidney waiting list, 2 pmp among those who were waiting for a liver transplant, 1 pmp among those who were waiting for a heart transplant, and less than 1 patient pmp among those who were on the waiting list for a lung transplant.

Table 1 provides additional descriptive statistics by consent system for the estimation sample of the main results for kidneys (for the full set of results for all organs, see Tables D–G in the S1 File). It shows that countries with different consent systems on average do not significantly differ from each other on these key variables. This holds for the estimation sample of the main results for each organ, except for hearts. In the estimation sample for the main results for hearts (column 3 of Table 2), countries with an opt-out system, on average, have significantly smaller populations and spend significantly less money on health per capita than those with an opt-in system.

**Table 1:** Descriptive statistics of key variables by consent system in the estimation sample for kidneys.

	(1)	(2)	(3)	(4)	(5)
	Total	Opt-in	Opt-out	Diff.	[*p*-value]
Population size (millions)	35.12	50.80	23.67	-27.13	[0.119]
	(57.46)	(78.68)	(32.10)		
Western countries (%)	53.33	47.37	57.69	10.32	[0.504]
	(50.45)	(51.30)	(50.38)		
Total deaths all causes (pmp, age-standardized)	54.45	51.48	56.12	4.64	[0.373]
	(14.78)	(10.99)	(16.54)		
GDP per capita (current US$/10,000)	21,434.12	20,983.99	21,763.06	779.07	[0.902]
	(20,552.31)	(17,540.11)	(22,839.46)		
Health expenditures per capita (current US$/1,000)	1,940.21	2,177.59	1,785.00	-392.59	[0.541]
	(2,024.45)	(2,189.79)	(1,937.27)		
Religious denomination (%)					
*None*	19.93	19.29	20.45	1.16	[0.857]
	(16.78)	(17.54)	(16.69)		
*Muslim*	9.74	11.29	8.47	-2.82	[0.743]
	(22.45)	(20.14)	(24.74)		
*Roman Catholic*	32.06	25.78	37.16	11.38	[0.338]
	(31.24)	(24.96)	(35.51)		
*Orthodox*	15.68	12.49	18.27	5.79	[0.624]
	(30.82)	(29.07)	(32.88)		
*Protestant*	6.43	6.80	6.13	-0.67	[0.892]
	(12.94)	(9.30)	(15.60)		
Number of countries	45	19	26		

Notes: Based on the estimation sample for the main results for kidneys (Table 2, column 1). See Tables D-G in the S1 File for the full set of descriptive statistics for the other organs.

**Table 2:** The relationship between consent systems and the number of transplantations from deceased donors (pmp) and the number of patients who died while on the waiting list (pmp), by organ.

		(1)	(2)	(3)	(4)
		Kidney	Liver	Heart	Lung
DDTX	α_1_: Opt-out (1 = yes)	7.082***	4.357***	1.031**	-0.106
		(2.331)	(1.258)	(0.430)	(0.678)
Mortality	β_1_: Opt-out (1 = yes)	-0.811	0.434	-0.171	-0.331*
		(1.423)	(0.494)	(0.280)	(0.197)
Chi^2^ test: α_1_+β_1_ = 0 [*p*-value]		[0.047]	[0.003]	[0.192]	[0.564]
Observations		549	514	492	390
Number of countries in the estimation sample		45	42	38	30

Notes: Each column presents the results of a seemingly unrelated OLS regression analysis. The dependent variables in each column are the number of transplantations from deceased donors per million population (DDTX) and the number of organ patients who died while on the waiting list per million population (Mortality). All regressions include year fixed effects, controls for total deaths (all causes), GDP per capita, health expenditures, religious denomination, and dummy variables for missing values on the independent variables. See Table P in the S1 File. We select countries that did not change their consent system after 1999, and we only include country-year observations for which both the number of transplantations from deceased donors and the number of waiting list deaths are non-missing. Robust standard errors clustered at the country level are reported in parentheses

*** p<0.01

** p<0.05

* p<0.1.

Source: Authors’ calculations based on Newsletter Transplant 2002–2016.

## 4. Results

### 4.1 Main results

Table 2 provides the main results. We first verify whether our data support the finding of other studies that countries with an opt-out consent system for organ donation have higher deceased-donor transplantation rates (i.e., conduct more transplantations from deceased donors pmp) than countries with opt-in systems. The results in the top panel of Table 2 show that this is indeed the case for kidneys, livers, and hearts. For lungs, we do not find a significant difference between opt-out and opt-in systems in deceased-donor transplantation rates. Deceased-donor kidney transplantation rates are on average 7.1 pmp higher in opt-out than in opt-in systems, there are 4.4 pmp more deceased-donor liver transplantations, and 1.0 pmp more deceased-donor hearts transplantations in opt-out relative to opt-in systems.

The second set of estimates in Table 2 shows the relationship between consent systems and organ-patient mortality rates (i.e., the number of patients who died while on a waiting list for organ transplantation pmp). For kidneys, livers and hearts, we find no significant difference regarding the number of patients who died while on the waiting lists (pmp) between consent systems. For lungs, we do find a significant difference: there are 0.3 fewer lung patients (pmp) who died while on the waiting list in opt-out consent systems than in opt-in systems.

However, the more important question is whether the difference between consent systems regarding organ-patient mortality rates is similar to what could be expected based on the difference in deceased-donor transplantation rates, i.e., whether if ${\hat{\alpha }}_{1}$+${\hat{\beta }}_{1}$ = 0. The answer to this question is given by the Chi^2^ tests, for which the *p*-values are given in the bottom of the table. The results show that, for kidneys and livers, the difference between opt-out and opt-in systems in terms of patient mortality rates is smaller than the difference in terms of deceased-donor transplantation rates. For hearts and lungs, on the other hand, the differences in terms of patient mortality and deceased-donor transplantation rates are similar, which is what one would expect if there is a one- to-minus-one relationship between deceased-donor transplantations and organ-patient mortality.

### 4.2 Potential mechanisms

The results in Table 2 indicate that consent systems do not affect kidney and liver-patient mortality rates through the deceased-donor transplantation rates alone. There must be other factors related to opt-out (opt-in) systems that increase (decrease) the number of deaths pmp on the waiting lists for kidneys and livers. In this section, we discuss in more detail the hypotheses on how differences in incentives between consent systems may explain our findings, and—where possible—we empirically analyze whether these hypotheses are likely to hold.

#### 4.2.1. Transplantations from living donors

One important example of potential differences in incentives between consent systems is that the larger supply of organs from deceased donors in opt-out systems may reduce people’s incentives to become a living donor. Considering that transplantations from living donors almost exclusively apply to kidneys and livers because living heart transplantations are not medically possible and living lung transplantations are still extremely rare, this is a particularly likely mechanism for our findings. Moreover, [4] and [13] already report evidence supporting the hypothesis that consent systems affect people’s incentives to become a living donor, as they find that countries with opt-out systems conduct fewer transplants from living donors than those with opt-in systems. Moreover, [14] show for the United States that an increase in the supply of cadaveric donors causes a decrease in the supply of living donors. This would mean that analyzing deceased-donor transplantation rates alone may overestimate the difference in transplantation rates between consent systems, and that the more accurate measure would be to analyze the *total* transplantation rates (i.e., deceased-donor plus living transplantations pmp). We can indeed analyze this with our data.

Similar to the result in our main analysis, Table 3 shows that the total transplantation rates are significantly higher in opt-out relative to opt-in systems. However, the difference between consent systems is smaller for the total kidney transplantation rate than for the deceased-donor kidney transplantation rate alone (i.e., 5.0 pmp compared to 7.1 pmp in Table 2). Moreover, now that the estimated opt-out effect on kidney transplantation rates is smaller, the estimated opt-out effect on kidney-patient mortality rates is no longer significantly different from it (*p* = 0.20 for kidneys). This means that the lower incentive to become a living kidney donor in opt-out systems (reflected by lower living kidney transplantation rates) can explain why kidney-patient mortality rates are not as low in opt-out systems as one would expect based on the deceased-donor kidney transplantation rates alone.

**Table 3:** The relationship between consent systems and the number of transplantations from deceased and living donors (pmp) and the number of patients who died while on the waiting list (pmp), by organ.

		(1)	(2)
		Kidney	Liver
Total TX	α_2_: Opt-out (1 = yes)	5.046**	5.211***
		(2.369)	(1.371)
Mortality	β_2_: Opt-out (1 = yes)	-0.784	0.485
		(1.405)	(0.495)
Chi^2^ test: α_2_+β_2_ = 0 [*p*-value]		[0.200]	[0.001]
Observations		534	485
Number of countries in the estimation sample		44	39

Notes: Each column presents the results of a seemingly unrelated OLS regression analysis. The dependent variables in each column are the number of transplantations from deceased donors plus the number of transplantations from living donors per million population (Total TX), and the number of organ patients who died while on the waiting list per million population (Mortality). All regressions include year fixed effects, controls for total deaths (all causes), GDP per capita, health expenditures, religious denomination, and dummy variables for missing values on the independent variables. See Table Q in the S1 File. We select countries that did not change their consent system after 1999, and we only include country-year observations for which both the number of transplantations from deceased donors, the number transplantations from living donors, and the number of waiting list deaths are non-missing. Robust standard errors clustered at the country level are reported in parentheses

*** p<0.01

** p<0.05

* p<0.1.

Source: Authors’ calculations based on Newsletter Transplant 2002–2016.

In contrast to the advantage of receiving a kidney from a living donor relative to receiving one from a deceased donor [15], live liver transplants have no mortality advantage over cadaveric liver transplants [16]. Therefore, the incentive to become a living liver donor is not the same as the incentive to become a living kidney donor. Our analysis indeed shows that the pattern we find for kidneys does not hold for livers. The point estimates, and the difference between the point estimates for the transplantation rates and the liver-patient mortality rates, remain similar to those in Table 2. It is therefore unlikely that liver transplantations from living donors are a mechanism for the surprisingly small differences in liver-patient mortality rates between consent systems.

#### 4.2.2. Participation in organ trade

The smaller deceased-donor and total supply of donor organs in opt-in systems creates incentives for organ-patients in countries with opt-in systems to try to speed up the process by purchasing an organ. However, despite the worldwide shortage of organs, the commercial trade of organs is illegal in almost all countries. When there is a black market for organ transplantations, the true number of transplantations in the donor’s country may be higher than what is reported in our data. At the same time, organ patients who consider purchasing their organ may not even enter the waiting list. If the purchase was successful in the sense that the recipient (who did not enter the waiting list) does not die, we overestimate the organ-patient mortality rates in the recipient’s country. However, if the (potential) recipient who did not enter the waiting list dies either before or after having purchased the organ, we underestimate the organ-patient mortality rates in the (potential) recipient’s country. Both mechanisms may lead to biased estimates of the opt-out effects on transplantation rates and organ-patient mortality rates.

Despite the illegality of commercial organ trade, there have been reports of organ sales in the following countries in our data (i.e., “organ-exporting countries”): Bolivia, Brazil, Colombia, Egypt, Iran, Moldova, Peru, and Turkey [17]. According to the same source, major organ-importing countries (i.e. the recipients’ countries of origin) included in our data are: Australia, Canada, Israel, and the United States. Paid kidney donation is legal in Iran, but there is strict regulation of the allocation of organs to non-local citizens, thereby restricting the international organ trade [17]. According to [4], there are also high incidences of organ trafficking in Ukraine.

In Table 4, we show the results when estimating the same regressions as in Table 2, while excluding this list of organ-exporting and organ-importing countries. Looking at the *p*-values in the bottom of the table, the conclusions are identical to those in Table 2: the difference between consent systems with regards to heart- and lung-patient mortality rates is similar to what could be expected based on the difference in deceased-donor transplantation rates, but the difference in kidney- and liver-patient mortality rates is significantly smaller (closer to zero) than the difference in deceased-donor transplantation rates. This implies that, although the incentive to purchase an organ on the black market may be higher in opt-in systems, there is no empirical evidence that this substantially affects deceased-donor transplantation rates or organ-patient mortality rates.

**Table 4:** The relationship between consent systems and the number of transplantations from deceased donors (pmp) and the number of patients who died while on the waiting list (pmp) in countries without reports of frequent organ trafficking, by organ.

		(1)	(2)	(3)	(4)
		Kidney	Liver	Heart	Lung
DDTX	α_3_: Opt-out (1 = yes)	9.333***	5.628***	1.350**	0.843
		(2.642)	(1.476)	(0.625)	(0.780)
Mortality	β_3_: Opt-out (1 = yes)	-1.975	0.766	-0.163	-0.200
		(1.855)	(0.493)	(0.375)	(0.152)
Chi^2^ test: α_3_+β_3_ = 0 [*p*-value]		[0.069]	[0.000]	[0.216]	[0.400]
Observations		423	398	382	292
Number of countries in the estimation sample		34	33	29	23

Notes: Each column presents the results of a seemingly unrelated OLS regression analysis. The dependent variables in each column are the number of transplantations from deceased donors per million population (DDTX) and the number of organ patients who died while on the waiting list per million population (Mortality). All regressions include year fixed effects, controls for total deaths (all causes), GDP per capita, health expenditures, religious denomination, and dummy variables for missing values on the independent variables. See Table R in the S1 File. We select countries that did not change their consent system after 1999, and we only include country-year observations for which both the number of transplantations from deceased donors and the number waiting list deaths are non-missing. Additionally, we exclude countries with reports of organ trafficking as either an organ-importing or organ-exporting country (i.e., Australia, Bolivia, Brazil, Canada, Colombia, Egypt, Iran, Israel, Moldova, Peru, Turkey, Ukraine and the United States). Robust standard errors clustered at the country level are reported in parentheses

*** p<0.01

** p<0.05, * p<0.1.

Source: Authors’ calculations based on Newsletter Transplant 2002–2016.

### 4.3 Robustness checks

This section discusses the results of a variety of robustness checks. As we established in Section 4.2 that it is inappropriate to focus on deceased-donor transplantation rates alone when comparing transplantation rates between consent systems, we focus on robustness checks of the results presented in Table 3, i.e., of the opt-out effect on the *total* transplantation rates and organ-patient mortality rates.

#### 4.3.1. Waiting list removals

Patients who are too sick to receive an organ may be taken off the waiting list (see, e.g., [18]). If this occurs less (more) often in opt-out systems than in opt-in systems, we will be underestimating (overestimating) the difference in the organ-patient mortality rates between the systems. We found information on the number of patients taken off the waiting list from various transplantation organizations for 16 of the countries in our data set. Although health deterioration is the main reason that patients are removed from a waiting list, patients may also be taken off the list for reasons other than being too sick. Examples of other registered reasons for waiting list removals are: “condition improved,” “recovered,” “not sick enough to receive a transplant,” “refused transplant,” and “other/unknown.” The data therefore provide an upper bound of the relation between consent systems and organ-patient mortality rates. Table H in the S1 File shows a list of the countries for which we have data on waiting list removals.

Table J in the S1 File shows that for this selection of countries and with this new measure of organ-patient mortality rates, the difference between consent systems with respect to kidney-patient mortality rates remains not significantly different from the difference in the total kidney transplantation rates. In other words: lower incentives to become a living kidney donor in opt-out systems remain a plausible mechanism for the surprisingly small difference in kidney-patient mortality rates between consent systems when waiting list removals are taken into account. For livers, the results remain robust to those shown in Table 3: when waiting list removals are taken into account, the estimated opt-out effect on total liver transplantation rates remains significantly different from the opt-out effect on liver-patient mortality rates.

#### 4.3.2. Length of the waiting list

By construction, there are fewer patients who can die while on the waiting list when waiting lists are shorter. Therefore, if waiting lists are generally longer (shorter) in opt-out systems, our estimated opt-out effects on organ-patient mortality rates will be biased. We measure waiting list length in year_t_ by taking the number of organ patients registered on the waiting list on December 31 in year_t-1_, and adding to this the number of patients who entered the waiting list throughout the current year_t_. These data are obtained from the Newsletters Transplant.

On average, the number of patients who receive a kidney transplant from either a living or deceased donor is equal to 29.2% of the number of patients registered on a kidney waiting list (see column 1 in Table K in the S1 File). In opt-out systems, these “relative kidney transplantation rates” are on average 1.3 percentage points higher than in opt-in systems (not statistically significant). For livers, the relative transplantation rates, as well as the differences between consent systems, are much larger, i.e., 45.6% and 12.2 percentage points, respectively (significant at the 5% level). On average, the number of patients who died while on a waiting list for kidney transplantation is equal to 3.6% of the number of patients who were on the kidney waiting list. For liver patients, the relative mortality rate is 9.0%.

The results in Table K in the S1 File indicate that the length of the waiting lists does not vary substantially between consent systems because for both kidneys and livers, the conclusions of Table 3 remain robust when taking waiting list length into account.

#### 4.3.3. Co-morbid conditions

Co-morbid conditions may affect the number of deaths on the waiting list, particularly for kidneys, since patients with end-stage renal disease can live for years on dialysis. Indeed, while organ patients are waiting for a donor organ, they might die from something else than organ failure, such as co-morbid conditions or even a traffic accident. That is why we control for countries’ general mortality rates in our analyses, thereby taking into account that in some countries, people—including organ patients—may be more likely to develop (fatal) co-morbid conditions than in others, and that this may systematically differ between consent systems.

This method works best if organ patients have a similar probability of dying from other factors than organ failure as their fellow countrymen. If organ patients have an increased probability of developing co-morbid conditions, controlling for general mortality rates might not be sufficient. It is important to keep in mind that this can only change our conclusions if the probability of having (fatal) co-morbid conditions is different between countries with opt-out and opt-in systems. The main reasons why one consent system would systematically have more kidney patients who die from co-morbid conditions than the other are (1) longer waiting time, e.g. due to smaller transplantation rates or more inefficient systems (note that, while kidney failure can be acute, the chance to suffer from co-morbid conditions will by construction increase as time goes by), and (2) unhealthier kidney patients are allowed to enter or remain on the waiting list, thereby increasing their probability to have or develop co-morbid conditions that ultimately become fatal. We do not have data to investigate these points, so we need to leave this point for future research.

#### 4.3.4. Dealing with zero values

Both the data on deceased-donor and living transplantations and on organ-patient mortality are heavily right-skewed with many zero values. This may pose two problems in the data. First, although we already set all country-organ observations to missing when only zeros and missing values are reported for that organ across years, it is possible that not all remaining zeros are “true zeros.” Some countries might, for instance, have reported a zero when there were no (reliable) data in a specific year. That means that there might be different mechanisms driving the increase from zero to one transplantation or death on the waiting list, than those driving the increase from one to two or infinity. We therefore run the same analyses as in Table 3, while excluding all zero values. Table L in the S1 File shows that the conclusions remain robust for this specification.

Second, because the data are censored at zero with many zero values, a Tobit regression might better fit our data than OLS. However, Table M in the S1 File shows that the conclusions remain robust for Tobit regressions.

#### 4.3.5. Country heterogeneity within consent systems

We also analyze whether the non-significant relationship between consent systems and organ-patient mortality rates is due to the potentially large heterogeneity in organ-patient mortality among countries that have the same consent system. To decrease heterogeneity within consent systems, we run the same analyses as in Table 3 while only including European countries, Australia, Canada, New Zealand, and the United States (i.e., excluding Latin American, Asian, and African countries). By doing so, the included countries are likely to be more homogenous with respect to unobservable characteristics that might affect transplantation and mortality rates. Table N in the S1 File shows that the patterns shown in Table 3 remain robust with this specification.

#### 4.3.6. Truncating the data

Finally, we analyze whether our results are driven by outliers in either the transplantation or the organ-patient mortality data. However, running the analyses after truncating both variables at the 99^th^ percentile leads to the same results (see Table O in the S1 File).

## 5. Conclusions

This paper analyzes the relationship between consent systems and the number of organ patients who died while waiting for a donor organ. Previous research has shown that countries with an opt-out consent system for organ donation conduct more deceased-donor transplantations than countries with an opt-in system. One might therefore expect that organ-patient mortality rates are lower in opt-out systems. We find that organ-patient mortality rates (i.e., the number of patients who died while on a waiting list, divided by the size of the population) among heart patients are indeed as low in countries with opt-out systems as could be expected based on the higher deceased-donor transplantation rates in these countries. For lungs, deceased-donor transplantation rates do not differ significantly between consent systems, and this is reflected in the mortality rates that are also not significantly different from each other. However, the difference between consent systems with respect to kidney- and liver-patient mortality rates is significantly different from the difference in deceased-donor transplantation rates. While deceased-donor kidney and liver transplantations are significantly higher in opt-out systems compared to opt-in systems, the number of patients (per million population) who died while on the waiting list for one of these organs is similar between consent systems. For kidneys, an explanation for this surprising result is that opt-out consent systems create fewer incentives to become living donors. Transplantations from living donors do not explain the findings for livers. These results are robust for various specifications.

Our results indicate that focusing on the deceased-donor transplantation rates alone paints an incomplete picture of the benefits of opt-out systems compared to opt-in systems. For kidneys and livers, consent systems for organ donation apparently do not affect organ-patient mortality rates through deceased-donor transplantation rates alone; there are other factors related to consent systems that affect the number of deaths for people on the kidney and liver waiting lists. We find that the number of living transplantations can explain the findings regarding kidneys, but not for livers. Waiting list removals and the length of the waiting lists appear to be unlikely mechanisms.

Moreover, it is worth noting that the number of kidney patients who died while on the waiting list is similar between consent systems, even though kidney transplantations from living donors are lower in opt-out systems compared to opt-in systems. Previous research shows that graft and patient survival rates are higher among those who received a kidney from a living donor than among those who received one from a deceased donor [15]. This indicates that living-donor kidney recipients may be less likely to re-enter the waiting list because their body rejected the donor kidney, and would therefore, by construction, be less likely to die while on a kidney waiting list. However, even if this were true, the impact on the number of deaths of people on the kidney waiting list must be minimal; otherwise, we would have found that the number of deaths among kidney patients (per million population) in opt-out systems is significantly higher than in opt-in systems. This is an important addition to the discussion on the benefits and drawbacks of the different consent systems.

More research is needed to identify determinants of organ-patient mortality rates. One potentially important topic for future research concerns the fact that countries with large organ shortages also have an incentive to participate in legal organ trade within a so-called “transplant network.” Examples are Eurotransplant or Scandiatransplant, where organs that cannot be allocated within the donor’s country because there is no matching recipient are allocated to a matching recipient from another country within the network. If countries with opt-in systems could acquire more organs (and particularly more kidneys and livers) through such networks than countries with opt-out systems, it might explain why the difference in kidney- and liver-patient mortality rates is so small between consent systems. Unfortunately, we cannot empirically test whether this hypothesis holds, because (1) almost every country in our data participates in a transplant network, either formally or through bilateral agreements, and (2) we do not have data on countries of origin of organ donors or recipients. A second potentially important topic for future research concerns investments in medical technology to decrease organ-patient mortality. The smaller supply of donor organs in countries with opt-in systems provides an incentive for these countries’ governments and researchers to invest in research and technology to increase organ-patients’ longevity or search for alternative sources of organs such as xenotransplants (i.e., transplanting animal organs into humans) or synthetic organs. If successful (for kidneys and livers), these investments would, in turn, decrease organ-patient mortality rates in opt-in systems, which would explain the surprisingly small differences between consent systems with respect to kidney- and liver-patient mortality rates. Although we do control for countries’ overall health expenditures, we unfortunately do not have data on the size of the investments in these specific medical technologies that may decrease organ-patient mortality nor on the share of the overall health expenditures that are allocated to them. It is therefore not possible to provide empirical evidence for the hypothesis that differences between consent systems with respect to the incentives for investing in organ-patient mortality-decreasing medical technology may explain our findings.

The main implication of our analyses is that it is not evident that opt-out consent systems reduce kidney and liver-patient mortality rates. For kidneys and livers—by far the most commonly transplanted organs—the higher deceased-donor transplantation rates in opt-out systems give a false impression that presumed consent is related to lower organ-patient mortality rates.

## Supporting information

S1 File Appendix. (DOCX)

## References

[1] Howard D. Producing organ donors. Journal of Economic Perspectives. 2007;21(3): 25–36. 10.1257/jep.21.3.25 19728420

[2] Kessler J, Roth A. Getting more organs for transplantation. American Economic Review: Papers and Proceedings. 2014;104(5): 425–430. 10.1257/aer.104.5.425 29115805

[3] Ugur Z. Does presumed consent save lives? Evidence from Europe. Health Economics. 2015;24(12): 1560–1572. 10.1002/hec.3111 25273232

[4] Shepherd L, O’Carroll R, Ferguson E. An international comparison of deceased and living organ donation/transplant rates in opt-in and opt-out systems: a panel study. BMC Medicine. 2014;12(131): 1–14. 10.1186/s12916-014-0131-4 25285666 PMC4175622

[5] Rithalia A, McDaid C, Suekarran S, Myers L, Sowden A. Impact of presumed consent for organ donation on donation rates: a systematic review. BMJ. 2009;338: 1–8. 10.1136/bmj.a3162 PMC2628300 19147479

[6] Abadie A, Gay S. The impact of presumed consent legislation on cadaveric organ donation: a cross-country study. Journal of Health Economics. 2006;25(4): 599–620. 10.1016/j.jhealeco.2006.01.003 16490267

[7] Johnson E, Goldstein D. Do defaults save lives? Science. 2003;302(5649): 1338–1339. 10.1126/science.1091721 14631022

[8] Johnson E, Goldstein D. Defaults and donation decisions. Transplantation. 2004;78(12): 1713–1716. 10.1097/01.tp.0000149788.10382.b2 15614141

[9] Dalen H, Henkens K. Comparing the effects of defaults in organ donation systems. Social Science and Medicine. 2014;106: 137–142. 10.1016/j.socscimed.2014.01.052 24561775

[10] Li D, Hawley Z, Schnier K. Increasing organ donation via changes in the default choice or allocation rule. Journal of Health Economics. 2013;32: 1117–1129. 10.1016/j.jhealeco.2013.09.007 24135615 PMC3855900

[11] Davidai S, Gilovich T, Ross L. The meaning of default options for potential organ donors. Proceedings of the National Academy of Sciences. 2012;109(38): 15201–15205. 10.1073/pnas.1211695109 22949639 PMC3458339

[12] Mossialos E, Costa-Font J, Rudisill C. Does organ donation legislation affect individuals’ willingness to donate their own or their relative’s organs? Evidence from European Union survey data. BMC Health Services Research. 2008;8(48): 1–10. 10.1186/1472-6963-8-48 18304341 PMC2292708

[13] Arshad A, Anderson B, Sharif A. Comparison of organ donation and transplantation rates between opt-out and opt-in systems. Kidney International. 2019;95(6): 1453–1460. 10.1016/j.kint.2019.01.036 31010718

[14] Fernandez J, Howard D, Stohr Kroese L. The effect of cadaveric kidney donations on living kidney donations: an instrumental variable approach. Economic Inquiry. 2013;51(3): 1696–1714.

[15] Nemati E, Einollahi B, Pezeshki ML, Porfarziani V, Fattahi MR. Does kidney transplantation with deceased or living donor affect graft survival? Nephro-Urology Monthly. 2012;6(4): e12182. 10.5812/numonthly.12182 PMC4317718 25695017

[16] Thuluvath PJ, Yoo HY. Graft and patient survival after adult live donor liver transplantation compared to a matched cohort who received a deceased donor transplantation. Liver Transplantation. 2004;10(10): 1263–1268. 10.1002/lt.20254 15376301

[17] Shimazono Y. The state of the international organ trade: a provisional picture based on integration of available information. Bulletin of the World Health Organization. 2007;85(12): 901–980. 10.2471/blt.06.039370 18278256 PMC2636295

[18] Charpentier K, Mavanur A. Removing patients from the liver transplant waiting list: a survey of US liver transplant programs. Liver Transplantation. 2008;14: 303–307. 10.1002/lt.21353 18306339

