# Supplementary material for: Deceased by default: Consent systems and organ-patient mortality
Source: PLoS One. 2021 Mar 17;16(3):e0247719. doi: 10.1371/journal.pone.0247719 (PMC7968695; doi:10.1371/journal.pone.0247719)
Supplement: S1 File — (DOCX) [file pone.0247719.s001.docx]

**S1 File. Appendix**

**Appendix 1. Data sources**

**1.1 Data sources for the main results**

**Organ-patient mortality rates and deceased-donor transplantation rates**

In every country, donated organs are scarce, and being on a waiting list for organ transplantation is usually the only way to legally receive an organ from a deceased donor. Therefore, being registered on the waiting list for organ transplantation is a good indicator of being an organ patient.

We use data on the number of patients who died while on the waiting list for a kidney, liver, heart, or lung transplantation from “Newsletter Transplant.” These newsletters also serve as the data source for the number of deceased-donor kidney, liver, heart, and lung transplantations^[[1]](#footnote-1)^ per country. The newsletters are published jointly by the Organicazión Nacional de Trasplantes (ONT) and the European Directorate for the Quality of Medicines & HealthCare of the Council of Europe (EDQM). National data in these documents are provided by representatives from national and international transplant networks.^[[2]](#footnote-2)^ The resulting data set contains information from 79 countries across a period of 15 years (2001–2015, unbalanced panel). We calculate organ-patient mortality and deceased-donor transplantation rates by dividing the absolute numbers by the country’s population size (in millions). Tables A and B list the description of the key variables and the countries included in the analyses. We define “organ patient mortality” as death while on the waiting list for a particular organ.

Table A. Description of key variables included in the analysis

| **Variable** | **Description** |
| --- | --- |
| Number of waiting list deaths | Number of patients who died while on the waiting list during the corresponding year. |
| Transplantations from deceased donors / deceased-donor transplantations | Transplantations from donors after brain death (i.e. death has been determined by neurological criteria) and donors after circulatory death (i.e. death has been determined by circulatory and respiratory criteria). Includes the transplantations of the corresponding organ with or without the simultaneous transplant of a different type of organ(s). Note that every donor can provide multiple organs, and that organs will not be removed from a potential donor for the purpose of transplantation unless there is a recipient. |

Source: Newsletter Transplant 2016

**Table B. List of countries included in the Newsletter Transplant for the first time**

| **Year** | **Countries** |
| --- | --- |
| 2001 | Australia, Austria, Belgium, Canada, Croatia, Cyprus, Czech Republic, Denmark, Estonia, Finland, France, Georgia, Germany, Greece, Hungary, Ireland, Israel, Italy, Kuwait, Latvia, Luxembourg, Malta, Netherlands, Norway, Poland, Portugal, Romania, Slovakia, Slovenia, Spain, Sweden, Switzerland, Turkey, United Kingdom, United States of America |
| 2002 | Bulgaria, Lithuania |
| 2003 | Argentina, Brazil, Bolivia, Chile, Colombia, Costa Rica, Cuba, Ecuador, El Salvador, Guatemala, Honduras, Iceland, Mexico, New Zealand, Panama, Paraguay, Peru, Puerto Rico, Dominican Republic, Uruguay, Venezuela |
| 2008 | Macedonia, Moldova, Nicaragua |
| 2010 | Algeria, Lebanon, Libya, Morocco, Palestine, Syria, Tunisia |
| 2011 | Belarus, Egypt, Russia |
| 2012 | Azerbaijan, Armenia, Ukraine |
| 2013 | Bosnia Herzegovina, Montenegro |
| 2014 | Iran, Malaysia, Sudan, Vietnam |

Source: Newsletter Transplant 2002–2016

Note: Puerto Rico is unincorporated territory of the United States and part of the United Network for Organ Sharing (UNOS).

**Consent systems**

We obtain data on the countries’ consent systems for organ donation from previous research articles, government websites, professional organizations, and legal documents (see Table C). For ten of the 79 countries, no (reliable) information on the consent system could be found. These countries are therefore excluded from the analysis.

We verify whether the countries’ consent system ever changed, and if so in which year, and adjust the opt-out variable accordingly. When no information is available for the year of introduction of the current consent system or whether the consent system changed between 2001 and 2015, we assume in our main analyses that the country has had the same consent system in all years. In our main analyses, we exclude countries that changed their consent system after 1999.

**Table C. Consent systems per country**

| Country | Current system | Since | Sources | | | |
| --- | --- | --- | --- | --- | --- | --- |
| Algeria | opt-in |  | *5* |  |  |  |
| Argentina | opt-out |  | *4* | *6* | *7* | *8* |
| Armenia | opt-in | 2002 | *3* |  |  |  |
| Australia | opt-in | 1980s | *3* | *4* | *9* |  |
| Austria | opt-out | 1982 | *2* | *3* | *4* |  |
| Azerbaijan | opt-out |  | *3* | *10* |  |  |
| Belarus | opt-out |  | *3* |  |  |  |
| Belgium | opt-out | 1986 | *2* | *3* | *4* | *9* |
| Bolivia | opt-in |  | *8* | *11* | *12* |  |
| Bosnia and Herzegovina | opt-out |  | *13* |  |  |  |
| Brazil | opt-in | 1997 | *3* | *4* | *7* | *8* |
| Bulgaria | opt-out | 2007 | *2* | *4* | *9* | *14* |
| Canada | opt-in | 1980s | *3* | *4* | *9* |  |
| Chile | opt-out |  | *3* | *4* |  |  |
| Colombia | opt-out |  | *3* | *4* | *8* |  |
| Costa Rica | opt-out | 1994 | *3* | *4* | *8* | *15* |
| Croatia | opt-out |  | *3* | *4* | *9* |  |
| Cuba | opt-in | 1988 | *3* | *4* | *8* |  |
| Cyprus | opt-out |  | *9* | *16* |  |  |
| Czech Republic | opt-out | 2002 | *3* | *4* | *9* |  |
| Denmark | opt-in | 1990 | *3* | *4* | *9* | *16* |
| Dominican | opt-out |  | *8* | *16* |  |  |
| Ecuador | opt-out | 1994 | *3* | *8* |  |  |
| Estonia | opt-out | 2002 | *2* | *4* | *9* | *16* |
| Finland | opt-out | 2001 | *3* | *9* |  |  |
| France | opt-out | 1976 | *3* | *4* | *9* |  |
| Georgia | opt-in |  | *4* | *14* |  |  |
| Germany | opt-in | 1997 | *3* | *9* |  |  |
| Greece | opt-out | 1999 | *4* | *9* |  |  |
| Guatemala | opt-in |  | *4* | *8* | *15* |  |
| Hungary | opt-out | 1997 | *4* | *9* |  |  |
| Iceland | opt-in | 1991 | *3* | *4* |  |  |
| Iran | opt-in |  | *18* |  |  |  |
| Ireland | opt-out | 2012 | *3* | *9* |  |  |
| Israel | opt-in |  | *3* | *4* | *9* |  |
| Italy | opt-out | 1999 | *3* | *9* |  |  |
| Latvia | opt-out | 1995 | *4* | *9* |  |  |
| Lithuania | opt-in | 1999 | *3* | *4* | *9* |  |
| Luxembourg | opt-out | 1982 | *3* | *4* | *9* |  |
| Macedonia | opt-out |  | *13* |  |  |  |
| Malaysia | opt-in | 1974 | *3* | *19* |  |  |
| Malta | opt-in |  | *3* | *4* | *20* |  |
| Mexico | opt-in |  | *3* | *8* |  |  |
| Moldova | opt-out |  | *4* | *21* |  |  |
| Montenegro | opt-in |  | *2* |  |  |  |
| Netherlands | opt-in | 1996 | *3* | *4* | *9* |  |
| New Zealand | opt-in |  | *3* | *4* | *9* |  |
| Norway | opt-out | 1973 | *3* | *9* | *16* |  |
| Panama | opt-in |  | *15* |  |  |  |
| Paraguay | opt-out | 1998 | *3* | *8* |  |  |
| Peru | opt-out |  | *8* |  |  |  |
| Poland | opt-out | 1995 | *3* | *4* | *9* |  |
| Portugal | opt-out | 1994 | *4* | *9* |  |  |
| Romania | opt-in |  | *3* | *9* | *16* |  |
| Russian F. | opt-out | 1992 | *3* | *4* |  |  |
| Slovakia | opt-out | 2004 | *3* | *4* | *9* |  |
| Slovenia | opt-out | 2000 | *3* | *9* |  |  |
| Spain | opt-out | 1979 | *3* | *4* | *9* |  |
| Sudan | opt-in |  | *22* | *23* |  |  |
| Sweden | opt-out | 1995 | *3* | *4* | *9* |  |
| Switzerland | opt-in | 2007 | *3* | *9* |  |  |
| Syria | opt-in |  | *23* |  |  |  |
| Turkey | opt-out | 1979 | *3* | *9* |  |  |
| United Kingdom | opt-in | 2006 | *3* | *4* | *9* |  |
| Ukraine | opt-in |  | *24* |  |  |  |
| Uruguay | opt-out |  | *25* |  |  |  |
| USA | opt-in |  | *3* | *4* | *9* |  |
| Venezuela | opt-in | 1992 | *3* | *4* | *8* |  |
| Vietnam | opt-in |  | *26* |  |  |  |

Note: Numbers in the table refer to the literature in the reference list.

**Confounders**

In our main specifications, we control for countries’ gross domestic product (GDP) per capita, health expenditures per capita, general mortality rates (all causes; age standardized), and religion. Table D provides descriptive statistics for these variables by consent system for kidneys. Tables E-G show the descriptive statistics for the samples used to estimate the opt-out effects for livers, hearts, and lungs. These tables show that there are no statistically significant differences in the characteristics between opt-in and opt-out countries.

**Table D. Means and standard deviations of key variables by consent system in the estimation sample for kidneys**

|  | (1) | (2) | (3) | (4) | (5) |
| --- | --- | --- | --- | --- | --- |
|  | Total | Opt-in | Opt-out | Diff. | [*p*-value] |
|  |  |  |  |  |  |
| Population size (millions) | 35.12 | 50.80 | 23.67 | -27.13 | [0.119] |
|  | (57.46) | (78.68) | (32.10) |  |  |
| Western countries (%) | 53.33 | 47.37 | 57.69 | 10.32 | [0.504] |
|  | (50.45) | (51.30) | (50.38) |  |  |
| Total deaths all causes (pmp, age-standardized) | 54.45 | 51.48 | 56.12 | 4.64 | [0.373] |
|  | (14.78) | (10.99) | (16.54) |  |  |
| GDP per capita (current US$/10,000) | 21,434.12 | 20,983.99 | 21,763.06 | 779.07 | [0.902] |
|  | (20,552.31) | (17,540.11) | (22,839.46) |  |  |
| Health expenditures per capita (current US$/1,000) | 1,940.21 | 2,177.59 | 1,785.00 | -392.59 | [0.541] |
|  | (2,024.45) | (2,189.79) | (1,937.27) |  |  |
| Religious denomination (%) |  |  |  |  |  |
| *None* | 19.93 | 19.29 | 20.45 | 1.16 | [0.857] |
|  | (16.78) | (17.54) | (16.69) |  |  |
| *Muslim* | 9.74 | 11.29 | 8.47 | -2.82 | [0.743] |
|  | (22.45) | (20.14) | (24.74) |  |  |
| *Roman Catholic* | 32.06 | 25.78 | 37.16 | 11.38 | [0.338] |
|  | (31.24) | (24.96) | (35.51) |  |  |
| *Orthodox* | 15.68 | 12.49 | 18.27 | 5.79 | [0.624] |
|  | (30.82) | (29.07) | (32.88) |  |  |
| *Protestant* | 6.43 | 6.80 | 6.13 | -0.67 | [0.892] |
|  | (12.94) | (9.30) | (15.60) |  |  |
| Missing values (%) |  |  |  |  |  |
| *Total deaths all causes* | 31.88 | 37.46 | 27.80 | -9.66 | [0.402] |
|  | (37.69) | (43.90) | (32.73) |  |  |
| *GDP per capita* | 2.22 | 5.26 | 0.00 | -5.26 | [0.037] |
|  | (8.41) | (12.49) | (0.00) |  |  |
| *Health expenditures per capita* | 4.44 | 10.53 | 0.00 | -10.53 | [0.095] |
|  | (20.84) | (31.53) | (0.00) |  |  |
| *Religious denomination* | 35.56 | 31.58 | 38.46 | 6.88 | [0.643] |
|  | (48.41) | (47.76) | (49.61) |  |  |
| Number of countries | 45 | 19 | 26 |  |  |

Notes: Based on the estimation sample for the main results for kidneys (Table 2, column 1).

**Table E. Means and standard deviations of key variables by consent system in the estimation sample for livers**

|  | (1) | (2) | (3) | (4) | (5) |
| --- | --- | --- | --- | --- | --- |
|  | Total | Opt-in | Opt-out | Diff. | [*p*-value] |
|  |  |  |  |  |  |
| Population size (millions) | 36.94 | 51.78 | 26.85 | -24.93 | [0.177] |
|  | (58.32) | (82.38) | (32.01) |  |  |
| Western countries (%) | 52.38 | 52.94 | 52.00 | -0.94 | [0.954] |
|  | (50.55) | (51.45) | (50.99) |  |  |
| Total deaths all causes (pmp, age-standardized) | 53.23 | 50.83 | 54.70 | 3.87 | [0.424] |
|  | (14.06) | (10.84) | (15.76) |  |  |
| GDP per capita (current US$/10,000) | 21,061.55 | 23,952.02 | 19,096.03 | -4,856.00 | [0.393] |
|  | (17,817.41) | (17,525.14) | (18,100.60) |  |  |
| Health expenditures per capita (current US$/1,000) | 1,974.20 | 2,526.18 | 1,643.02 | -883.16 | [0.168] |
|  | (1,947.62) | (2,198.52) | (1,743.49) |  |  |
| Religious denomination (%) |  |  |  |  |  |
| *None* | 21.66 | 21.54 | 21.74 | 0.19 | [0.974] |
|  | (15.66) | (16.84) | (15.26) |  |  |
| *Muslim* | 8.08 | 7.60 | 8.43 | 0.83 | [0.916] |
|  | (21.23) | (16.89) | (24.36) |  |  |
| *Roman Catholic* | 35.24 | 26.79 | 41.33 | 14.54 | [0.200] |
|  | (30.85) | (24.08) | (34.30) |  |  |
| *Orthodox* | 13.12 | 12.50 | 13.57 | 1.07 | [0.919] |
|  | (28.32) | (29.06) | (28.61) |  |  |
| *Protestant* | 6.36 | 6.80 | 6.05 | -0.75 | [0.873] |
|  | (12.57) | (9.30) | (14.74) |  |  |
| Missing values (%) |  |  |  |  |  |
| *Total deaths all causes* | 25.92 | 25.57 | 26.15 | 0.58 | [0.956] |
|  | (32.65) | (36.02) | (30.92) |  |  |
| *GDP per capita* | 0.79 | 1.96 | 0.00 | -1.96 | [0.082] |
|  | (3.59) | (5.54) | (0.00) |  |  |
| *Health expenditures per capita* | 4.76 | 11.76 | 0.00 | -11.76 | [0.082] |
|  | (21.55) | (33.21) | (0.00) |  |  |
| *Religious denomination* | 26.19 | 23.53 | 28.00 | 4.47 | [0.754] |
|  | (44.50) | (43.72) | (45.83) |  |  |
| Number of countries | 42 | 17 | 25 |  |  |

Notes: Based on the estimation sample for the main results for livers (Table 2, column 2).

**Table F. Means and standard deviations of key variables by consent system in the estimation sample for hearts**

|  | (1) | (2) | (3) | (4) | (5) |
| --- | --- | --- | --- | --- | --- |
|  | Total | Opt-in | Opt-out | Diff. | [*p*-value] |
|  |  |  |  |  |  |
| Population size (millions) | 40.73 | 63.73 | 27.31 | -36.42 | [0.076] |
|  | (61.05) | (88.47) | (32.60) |  |  |
| Western countries (%) | 60.53 | 64.29 | 58.33 | -5.95 | [0.726] |
|  | (49.54) | (49.72) | (50.36) |  |  |
| Total deaths all causes (pmp, age-standardized) | 53.34 | 50.98 | 54.51 | 3.53 | [0.500] |
|  | (13.90) | (11.61) | (15.03) |  |  |
| GDP per capita (current US$/10,000) | 21,936.88 | 25,755.68 | 19,709.25 | -6,046.44 | [0.329] |
|  | (18,173.39) | (18,138.93) | (18,201.22) |  |  |
| Health expenditures per capita (current US$/1,000) | 2,101.86 | 2,920.15 | 1,692.72 | -1,227.43 | [0.080] |
|  | (1,983.02) | (2,217.43) | (1,763.07) |  |  |
| Religious denomination (%) |  |  |  |  |  |
| *None* | 22.38 | 21.94 | 22.67 | 0.73 | [0.916] |
|  | (16.42) | (18.18) | (15.80) |  |  |
| *Muslim* | 7.08 | 6.57 | 7.42 | 0.85 | [0.924] |
|  | (21.36) | (15.01) | (25.23) |  |  |
| *Roman Catholic* | 37.20 | 26.03 | 44.65 | 18.62 | [0.155] |
|  | (31.75) | (23.55) | (34.97) |  |  |
| *Orthodox* | 9.08 | 9.32 | 8.92 | -0.40 | [0.968] |
|  | (24.23) | (26.92) | (23.24) |  |  |
| *Protestant* | 7.02 | 7.92 | 6.42 | -1.50 | [0.797] |
|  | (13.85) | (10.34) | (16.09) |  |  |
| Missing values (%) |  |  |  |  |  |
| *Total deaths all causes* | 25.76 | 27.50 | 24.74 | -2.76 | [0.810] |
|  | (33.45) | (39.59) | (30.18) |  |  |
| *GDP per capita* | 2.19 | 5.95 | 0.00 | -5.95 | [0.043] |
|  | (8.81) | (14.03) | (0.00) |  |  |
| *Health expenditures per capita* | 5.26 | 14.29 | 0.00 | -14.29 | [0.059] |
|  | (22.63) | (36.31) | (0.00) |  |  |
| *Religious denomination* | 34.21 | 28.57 | 37.50 | 8.93 | [0.588] |
|  | (48.08) | (46.88) | (49.45) |  |  |
| Number of countries | 38 | 14 | 24 |  |  |

Notes: Based on the estimation sample for the main results for hearts (Table 2, column 3).

**Table G. Means and standard deviations of key variables by consent system in the estimation sample for lungs**

|  | (1) | (2) | (3) | (4) | (5) |
| --- | --- | --- | --- | --- | --- |
|  | Total | Opt-in | Opt-out | Diff. | [*p*-value] |
|  |  |  |  |  |  |
| Population size (millions) | 46.81 | 67.93 | 32.74 | -35.19 | [0.159] |
|  | (66.47) | (94.09) | (35.83) |  |  |
| Western countries (%) | 66.67 | 66.67 | 66.67 | 0.00 | [1.000] |
|  | (47.95) | (49.24) | (48.51) |  |  |
| Total deaths all causes (pmp, age-standardized) | 51.87 | 50.14 | 52.92 | 2.79 | [0.608] |
|  | (13.84) | (11.90) | (15.13) |  |  |
| GDP per capita (current US$/10,000) | 26,740.60 | 30,775.91 | 24,050.40 | -6,725.51 | [0.328] |
|  | (18,124.07) | (16,518.04) | (19,095.73) |  |  |
| Health expenditures per capita (current US$/1,000) | 2,474.96 | 3,033.90 | 2,102.33 | -931.56 | [0.220] |
|  | (2,011.66) | (2,178.10) | (1,861.74) |  |  |
| None | 24.22 | 24.94 | 23.67 | -1.26 | [0.858] |
|  | (16.21) | (16.61) | (16.56) |  |  |
| Muslim | 8.21 | 7.77 | 8.56 | 0.79 | [0.938] |
|  | (23.28) | (18.66) | (27.06) |  |  |
| Roman Catholic | 34.93 | 27.16 | 40.91 | 13.74 | [0.304] |
|  | (31.11) | (22.48) | (36.14) |  |  |
| Orthodox | 6.14 | 0.75 | 10.29 | 9.54 | [0.240] |
|  | (18.95) | (0.92) | (24.80) |  |  |
| Protestant | 7.13 | 7.75 | 6.65 | -1.10 | [0.860] |
|  | (14.39) | (10.42) | (17.25) |  |  |
| Missing (total deaths all causes) | 19.00 | 16.65 | 20.58 | 3.93 | [0.662] |
|  | (23.53) | (27.14) | (21.47) |  |  |
| Missing (GDP) | 0.00 | 0.00 | 0.00 |  |  |
|  | (0.00) | (0.00) | (0.00) |  |  |
| Missing (health expenditures) | 0.00 | 0.00 | 0.00 |  |  |
|  | (0.00) | (0.00) | (0.00) |  |  |
| Missing (religious denomination) | 23.33 | 16.67 | 27.78 | 11.11 | [0.498] |
|  | (43.02) | (38.92) | (46.09) |  |  |
| Number of countries | 30 | 12 | 18 |  |  |

Notes: Based on the estimation sample for the main results for hearts (Table 2, column 4).

The GDP of a country is known to be a potential confounder in the relationship between consent systems and deceased-donor transplantation rates (e.g. Rithalia et al. 2009). It is likely to be related to organ-patient mortality rates because economically developed countries may, for example, be more able to invest in life-extending medical equipment. We obtain data on countries’ annual GDP per capita (in current US$) from the World Bank.^[[3]](#footnote-3)^

Besides GDP, health spending is also an indicator of a country’s wealth, life expectancy, and access to advanced medical equipment. Countries’ health expenditures may therefore be related to organ patient mortality as well as their transplantation capacity. Moreover, previous research has found some differences between consent systems with respect to health expenditures per capita (e.g., Abadie & Gay 2006). We obtain data on countries’ annual health expenditures per capita (in current US$) from the global health expenditure database from the World Health Organization (WHO).^[[4]](#footnote-4)^

It is important to control for general mortality rates, because, similar to the rest of the population, organ patients face the risk of dying from another cause besides organ failure. Moreover, previous research has shown that mortality rates affect deceased-donor transplantation rates (see, e.g., Coppen et al. 2005). Annual data on countries’ age-standardized death rates per 100,000 world standard population (total deaths, all causes) are obtained from the WHO mortality database.^[[5]](#footnote-5)^

Previous research has shown that deceased-donor transplantation rates and people’s willingness to donate are related to religion (e.g. Gimbel et al. 2003). More specifically, it has been suggested that Catholicism may be associated with favorable attitudes towards organ donation, because the religion officially recognizes organ transplantation as a “service of life” (Rithalia et al. 2009). We obtain country-level data on people’s religious denomination from the World Values Survey. This survey is conducted every five years, using a common questionnaire. It is nationally representative of all people aged 18 and older (regardless of their nationality, citizenship, or language) residing within private households. Unfortunately, not every country is included in each wave. To minimize the amount of missing religiosity data, we calculate each country’s average share of each religious denomination across waves 4–6 (i.e., 2000–2014). We can do this because religious denominations are relatively stable over time at the country level. In our analyses, we control for the percentage of people in a country who consider themselves to be part of the Roman Catholic denomination.

**1.2 Data sources for the mechanism section**

**Transplantations from living donors**

We obtain data on the number of kidney and liver transplantations from living donors from the Newsletter Transplant (2001–2015, unbalanced panel). Living heart transplantations are medically not possible and living lung transplantations (i.e., two living donors each provide a lobe to one recipient) are extremely rare.

**Removals from the waiting list**

The number of patients who died while on a waiting list does not necessarily reveal the total number of patients who die due to organ failure because patients who become too sick to be transplanted are often taken off the waiting list (see, e.g., Charpentier and Mavanur 2008). The probability that these patients die due to organ failure after being taken off the waiting list is high. Therefore, in Section 4.2 on Mechanisms, we use additional data on waiting list removals, which we obtain from various transplantation organizations, for a selection of 16 countries (see Table H). Although the main reason for waiting list removal is that patients became unfit for transplantation due to health deterioration, patients may also be taken off the list for other reasons. The data on waiting list removals therefore provide an upper bound of the number of deaths after being taken off the waiting list.

**Table H. Countries for which information on removals from the waiting list is available**

| Australia (2010–2016), Austria (2007–2016), Belgium (2007–2016), Croatia (2007–2016), Denmark (2011–2016), France (2010–2015), Finland (2011–2016), Germany (2007–2016), Hungary (2007–2016), Netherlands (2007–2016), New Zealand (2010–2016), Norway (2011–2016), Slovenia (2007–2016), Sweden (2011–2016), United Kingdom (2015), USA (2007–2016). |
| --- |

Sources: (*27, 28, 29, 30, 31, 32*).

**Length of the waiting list**

We calculate the relative organ-patient mortality rates and deceased-donor transplantation rates by dividing these variables by the number of patients on the waiting list on December 31 in the previous year (per million population) plus the number of patients who entered the waiting list throughout the year. These data are from the Newsletter Transplant (2001–2015, unbalanced panel).

**Appendix 2. Data selection**

Due to lack of (reliable) information on the national consent system for organ donation, we had to exclude Egypt, El Salvador, Honduras, Lebanon, Libya, Nicaragua, Palestine, Serbia, and Tunisia from all analyses. Puerto Rico is part of UNOS and deleted to avoid double counting.

In our main specifications, we select countries that did not change their consent system after 1999, because including them likely leads to biased estimates of the opt-out effects on transplantation rates and organ-patient mortality rates. Additionally, we only include country-year observations for which both the number of deceased-donor transplantations and the number of waiting list deaths are non-missing. By doing so, we ensure the same estimation sample for both outcomes, which improves the comparability of the results.

The data on transplantations and waiting list deaths contain many zeros and missing values. Although for some countries it may be true that in some years nobody died while on a waiting list or there were no transplantations, it is unclear whether a reported zero is a “true zero” when data for that country are missing in all other years. We therefore set all values of variable *y* for organ *i* in country *c* to missing when only zeros and missing values are reported.

We also replaced extreme outliers by the average value of variable *y* for organ *i* in country *c*. As a robustness check in the analyses, we truncate variables at the 99^th^ percentile for each organ separately.

The data on our control variables do not always cover all countries and years in our sample. We therefore create a dummy variable for each control variable, which has a value of 1 if country *c* has a missing value on control variable *x* in year *t*, and 0 otherwise, and we replace the missing value in the control variables with the value 0. In all regressions in which we include control variable *x¸* we also include the corresponding missing dummy.

In one of the robustness checks, we exclude countries with reports of high levels of organ trafficking. Among the countries included in our data, there have been reports of high incidences of organ trafficking, either as an organ-importing or organ-exporting country, in Australia, Bolivia, Brazil, Canada, Colombia, Egypt, Iran, Israel, Moldova, Peru, Turkey, Ukraine, and the United States (Shimazono, 2007; Shepherd, O’Carroll & Ferguson, 2014).

**Table I. Additional information for countries excluded from the sample**

| **Countries** | **Additional information** |
| --- | --- |
| Egypt, El Salvador, Honduras, Lebanon, Libya, Nicaragua, Palestine, Serbia, Tunisia | Excluded from all analyses due to lack of (reliable) information on the national consent system for organ donation (*4*). |
| Bulgaria, Czech Republic, Estonia, Finland, Ireland, Slovakia, Slovenia, Switzerland, United Kingdom^[[6]](#footnote-6)^ | Changed their consent system after 1999 (*2-3*). Excluded from the sample in the main analyses, but included in the sample as a robustness check |
| Australia, Bolivia, Brazil, Canada, Colombia, Egypt^[[7]](#footnote-7)^, Iran, Israel, Moldova, Peru, Turkey, Ukraine, USA | Reports of high levels of organ trafficking as either an organ-importing or organ-exporting country (*4, 38*). Included in the sample in the main analyses, but excluded from the sample as a robustness check. |
| Algeria, Argentina, Armenia, Azerbaijan, Belarus, Bolivia, Bosnia Herzegovina, Brazil, Chile, Colombia, Costa Rica, Cuba, Dominican Republic, Ecuador, Egypt,^7^ El Salvador,^7^ Georgia, Guatemala, Honduras,^7^ Iran, Israel, Kuwait, Lebanon, Libya, Macedonia, Malaysia, Mexico, Moldova, Montenegro, Morocco, Nicaragua,^7^ Palestine,^7^ Panama, Paraguay, Peru, Russia, Serbia,^7^ Sudan, Syria, Tunisia,^7^ Ukraine, Uruguay, Venezuela, Vietnam | Non-western countries (Latin American, Asian and African countries). Excluded from the sample as a robustness check. |

Note: Puerto Rico is part of UNOS and only listed as a separate entity in Newsletter Transplant 2003. In order to avoid double counting, it is excluded from all analyses.

**Appendix 3. Additional tables**

**Table J. The relationship between consent systems and the number of transplantations from deceased and living donors (pmp), and the number of patients who died while on the waiting list plus the number of removals from the waiting list (pmp), by organ**

|  |  | (1) | (2) |
| --- | --- | --- | --- |
|  |  | Kidney | Liver |
|  |  |  |  |
| Total TX | α_4_: Opt-out (1 = yes) | 5.148 | 7.032*** |
|  |  | (4.381) | (2.572) |
|  | Total deaths all causes (pmp, age-standardized) | -0.148 | -0.243* |
|  |  | (0.162) | (0.141) |
|  | GDP pc (current US$/10,000) | -2.518 | -4.141*** |
|  |  | (1.532) | (1.386) |
|  | Health expenditures pc (current US$/1,000) | 4.484*** | 3.390*** |
|  |  | (1.139) | (0.721) |
|  | Religious denomination (% Roman Catholic) | -0.040 | -0.200 |
|  |  | (0.164) | (0.131) |
|  | Constant | 38.442** | 28.220** |
|  |  | (17.823) | (11.742) |
| Mortality incl. removals | β_4_: Opt-out (1 = yes) | -3.550 | 1.038 |
|  |  | (2.352) | (1.660) |
|  | Total deaths all causes (pmp, age-standardized) | 0.138 | -0.149** |
|  |  | (0.117) | (0.072) |
|  | GDP pc (current US$/10,000) | -6.253*** | -2.961*** |
|  |  | (1.100) | (0.692) |
|  | Health expenditures pc (current US$/1,000) | 6.867*** | 2.590*** |
|  |  | (0.759) | (0.390) |
|  | Religious denomination (% Roman Catholic) | -0.016 | 0.043 |
|  |  | (0.102) | (0.059) |
|  | Constant | 3.030 | 12.601* |
|  |  | (10.787) | (6.461) |
| Chi^2^ test: α_4_+β_4_=0 [*p*-value] | | [0.807] | [0.044] |
|  | |  |  |
| Observations | | 101 | 100 |
| Number of countries in the estimation sample | | 12 | 13 |
|  | |  |  |
| Mean total TX rate in estimation sample | | 44.088 | 15.241 |
| *Standard deviation* | | *9.675* | *6.895* |
| Mean organ-patient mortality rate incl. waiting list removals in estimation sample | | 10.617 | 5.441 |
| *Standard deviation* | | *8.712* | *3.428* |

Notes: Each column presents the results of a seemingly unrelated OLS regression analysis. The dependent variables in each column are the number of transplantations from deceased and living donors per million population (Total TX) and the number of organ patients who died while on the waiting list per million population plus the number of patients who were removed from the waiting list per million population (Mortality includes removals). All regressions include year fixed effects and dummy variables for missing values on the independent variables. We select countries that did not change their consent system after 1999, and we only include country-year observations for which both the number of transplantations from deceased donors, the number of transplantations from living donors, the number of waiting list deaths, and the number of removals from the waiting lists are non-missing. Robust standard errors clustered at the country level are reported in parentheses, *** p<0.01, ** p<0.05, * p<0.1.

Source: Authors’ calculations based on Newsletter Transplant 2002-2016.

**Table K. The relationship between consent systems and the number of transplantations from deceased and living donors (pmp) and the number of patients who died while on the waiting list (pmp), relative to the length of the waiting lists, by organ**

|  |  | (1) | (2) |
| --- | --- | --- | --- |
|  |  | Kidney | Liver |
|  |  |  |  |
| Relative total TX | α_5_: Opt-out (1= yes) | 0.013 | 0.122** |
|  |  | (0.044) | (0.058) |
|  | Total deaths all causes (pmp, age-standardized) | 0.001 | -0.001 |
|  |  | (0.001) | (0.003) |
|  | GDP pc (current US$/10,000) | 0.092*** | 0.128*** |
|  |  | (0.019) | (0.026) |
|  | Health expenditures pc (current US$/1,000) | -0.060*** | -0.077*** |
|  |  | (0.012) | (0.022) |
|  | Religious denomination (% Roman Catholic) | -0.000 | 0.001 |
|  |  | (0.001) | (0.001) |
|  | Constant | 0.101 | 0.269 |
|  |  | (0.109) | (0.172) |
| Relative mortality | β_5_: Opt-out (1 = yes) | -0.006 | -0.017 |
|  |  | (0.006) | (0.013) |
|  | Total deaths all causes (pmp, age-standardized) | -0.000 | -0.000 |
|  |  | (0.000) | (0.001) |
|  | GDP pc (current US$/10,000) | -0.002 | -0.012** |
|  |  | (0.004) | (0.005) |
|  | Health expenditures pc (current US$/1,000) | 0.001 | -0.002 |
|  |  | (0.004) | (0.004) |
|  | Religious denomination (% Roman Catholic) | -0.000 | -0.000* |
|  |  | (0.000) | (0.000) |
|  | Constant | 0.037** | 0.179*** |
|  |  | (0.016) | (0.033) |
| Chi^2^ test: α_5_+β_5_=0 [*p*-value] | | [0.863] | [0.078] |
|  | |  |  |
| Observations | | 360 | 312 |
| Number of countries in the estimation sample | | 42 | 36 |
|  | |  |  |
| Mean relative total TX rate in estimation sample | | 0.292 | 0.456 |
| *Standard deviation* | | *0.254* | *0.237* |
| Mean relative organ-patient mortality rate in estimation sample | | 0.036 | 0.090 |
| *Standard deviation* | | *0.044* | *0.061* |

Notes: Each column presents the results of a seemingly unrelated OLS regression analysis. The dependent variables in each column are the number of transplantations from deceased and living donors per million population divided by the number of patients on the waiting list on December 31 of the previous year plus the number of new waiting list entrants throughout the current year per million population (Relative total TX), and the number of organ patients who died while on the waiting list per million population divided by the number of patients on the waiting list on December 31 of the previous year plus the number of new waiting list entrants throughout the current year per million population (Relative mortality). All regressions include year fixed effects and dummy variables for missing values on the independent variables. We select countries that did not change their consent system after 1999, and we only include country-year observations for which both the relative total transplantation rates and the relative mortality rates are non-missing. Robust standard errors clustered at the country level are reported in parentheses, *** p<0.01, ** p<0.05, * p<0.1.

Source: Authors’ calculations based on Newsletter Transplant 2002-2016.

**Table L. The relationship between consent systems and the number of transplantations from deceased and living donors (pmp) and the number of patients who died while on the waiting list (pmp), excluding zero values on the dependent variables, by organ**

|  |  | (1) | (2) |
| --- | --- | --- | --- |
|  |  | Kidney | Liver |
|  |  |  |  |
| Total TX | α_6_ : Opt-out (1 = yes) | 5.844** | 5.108*** |
|  |  | (2.289) | (1.336) |
|  | Total deaths all causes (pmp, age-standardized) | 0.098 | -0.111 |
|  |  | (0.094) | (0.075) |
|  | GDP pc (current US$/10,000) | 1.186 | -0.637 |
|  |  | (1.729) | (1.029) |
|  | Health expenditures pc (current US$/1,000) | 4.102*** | 2.661*** |
|  |  | (1.304) | (0.679) |
|  | Religious denomination (% Roman Catholic) | -0.069 | 0.025 |
|  |  | (0.047) | (0.031) |
|  | Constant | 16.796*** | 8.533* |
|  |  | (6.371) | (4.525) |
| Mortality | β_6_: Opt-out (1 = yes) | -0.738 | 0.437 |
|  |  | (1.457) | (0.493) |
|  | Total deaths all causes (pmp, age-standardized) | 0.008 | -0.020 |
|  |  | (0.031) | (0.017) |
|  | GDP pc (current US$/10,000) | -2.356*** | -1.056*** |
|  |  | (0.881) | (0.294) |
|  | Health expenditures pc (current US$/1,000) | 2.342*** | 0.991*** |
|  |  | (0.821) | (0.238) |
|  | Religious denomination (% Roman Catholic) | -0.035 | -0.012 |
|  |  | (0.031) | (0.013) |
|  | Constant | 4.987* | 3.668** |
|  |  | (2.733) | (1.436) |
| Chi^2^ test: α_6_+β_6_=0 [*p*-value] | | [0.124] | [0.001] |
|  |  |  |  |
| Observations | | 518 | 469 |
| Number of countries in the estimation sample | | 44 | 39 |
|  |  |  |  |
| Mean total TX rate in estimation sample | | 28.915 | 9.852 |
| *Standard deviation* | | *15.621* | *7.452* |
| Mean organ-patient mortality rate in estimation sample | | 10.617 | 5.441 |
| *Standard deviation* | | *8.712* | *3.428* |

Notes: Each column presents the results of a seemingly unrelated OLS regression analysis. The dependent variables in each column are the number of transplantations from deceased and living donors per million population (Total TX), and the number of organ patients who died while on the waiting list per million population (Mortality). All regressions include year fixed effects and dummy variables for missing values on the independent variables. We select countries that did not change their consent system after 1999, and we only include country-year observations for which both the total transplantation rates and the organ-patient mortality rates are non-missing. Additionally, we exclude zero values on the dependent variables. Robust standard errors clustered at the country level are reported in parentheses, *** p<0.01, ** p<0.05, * p<0.1.

Source: Authors’ calculations based on Newsletter Transplant 2002-2016.

**Table M. The relationship between consent systems and the number of transplantations from deceased and living donors (pmp) and the number of patients who died while on the waiting list (pmp), by organ. Marginal effects after Tobit regressions.**

|  |  | (1) | (2) |
| --- | --- | --- | --- |
|  |  | Kidney | Liver |
|  |  |  |  |
| Total TX | α_7_ : Opt-out (1 = yes) | 4.921** | 4.706*** |
|  |  | (2.315) | (1.225) |
|  | Total deaths all causes (pmp, age-standardized) | 0.005 | -0.100* |
|  |  | (0.132) | (0.058) |
|  | GDP pc (current US$/10,000) | 1.556 | -0.668 |
|  |  | (1.798) | (0.866) |
|  | Health expenditures pc (current US$/1,000) | 3.691*** | 2.494*** |
|  |  | (1.411) | (0.575) |
|  | Religious denomination (% Roman Catholic) | -0.043 | 0.024 |
|  |  | (0.052) | (0.026) |
| Mortality | β_7_: Opt-out (1 = yes) | -0.614 | 0.429 |
|  |  | (1.102) | (0.437) |
|  | Total deaths all causes (pmp, age-standardized) | -0.001 | -0.023 |
|  |  | (0.026) | (0.016) |
|  | GDP pc (current US$/10,000) | -1.783** | -0.934*** |
|  |  | (0.709) | (0.253) |
|  | Health expenditures pc (current US$/1,000) | 1.809*** | 0.894*** |
|  |  | (0.653) | (0.207) |
|  | Religious denomination (% Roman Catholic) | -0.024 | -0.008 |
| Chi^2^ test: α_7_+β_7_=0 [*p*-value] | | [0.200] | [0.001] |
|  |  |  |  |
| Observations | | 534 | 485 |
| Number of countries in the estimation sample | | 44 | 39 |
|  |  |  |  |
| Mean total TX rate in estimation sample | | 28.270 | 9.632 |
| *Standard deviation* | | *15.879* | *7.482* |
| Mean organ-patient mortality rate in estimation sample | | 4.618 | 2.026 |
| *Standard deviation* | | *5.906* | *1.711* |

Notes: Each column presents the marginal effects obtained after a seemingly unrelated left-censored Tobit regression analysis. The dependent variables in each column are the number of transplantations from deceased donors per million population plus the number of transplantations from living donors per million population (Total TX), and the number of organ patients who died while on the waiting list per million population (Mortality). All regressions include year fixed effects and dummy variables for missing values on the independent variables. We select countries that did not change their consent system after 1999, and we only include country-year observations for which both the number of transplantations from deceased donors, the number transplantations from living donors, and the number of waiting list deaths are non-missing. Robust standard errors clustered at the country level are reported in parentheses, *** p<0.01, ** p<0.05, * p<0.1.

Source: Authors’ calculations based on Newsletter Transplant 2002-2016.

**Table N. The relationship between consent systems and the number of transplantations from deceased and living donors (pmp) and the number of patients who died while on the waiting list (pmp), excluding non-Western countries, by organ**

|  |  | (1) | (2) |
| --- | --- | --- | --- |
|  |  | Kidney | Liver |
|  |  |  |  |
| Total TX | α_8_: Opt-out (1 = yes) | 5.553** | 6.433*** |
|  |  | (2.510) | (1.357) |
|  | Total deaths all causes (pmp, age-standardized) | -0.032 | -0.331*** |
|  |  | (0.137) | (0.071) |
|  | GDP pc (current US$/10,000) | -1.083 | -2.992*** |
|  |  | (1.381) | (0.920) |
|  | Health expenditures pc (current US$/1,000) | 4.154*** | 3.312*** |
|  |  | (1.062) | (0.784) |
|  | Religious denomination (% Roman Catholic) | -0.106** | 0.033 |
|  |  | (0.041) | (0.032) |
|  | Constant | 28.739*** | 22.291*** |
|  |  | (8.706) | (4.358) |
| Mortality | β_8_: Opt-out (1 = yes) | -1.485 | 0.758 |
|  |  | (1.516) | (0.589) |
|  | Total deaths all causes (pmp, age-standardized) | -0.036 | -0.039* |
|  |  | (0.041) | (0.023) |
|  | GDP pc (current US$/10,000) | -3.375*** | -1.593*** |
|  |  | (0.889) | (0.252) |
|  | Health expenditures pc (current US$/1,000) | 2.494*** | 1.208*** |
|  |  | (0.791) | (0.199) |
|  | Religious denomination (% Roman Catholic) | -0.070*** | -0.011 |
|  |  | (0.017) | (0.014) |
|  | Constant | 10.581*** | 5.128*** |
|  |  | (3.092) | (1.864) |
| Chi^2^ test: α_8_+β_8_=0 [*p*-value] | | [0.245] | [0.000] |
|  |  |  |  |
| Observations | | 337 | 309 |
| Number of countries in the estimation sample | | 23 | 21 |
|  |  |  |  |
| Mean total TX rate in estimation sample | | 35.803 | 13.154 |
| *Standard deviation* | | *13.463* | *3.428* |
| Mean organ-patient mortality rate in estimation sample | | 10.617 | 5.441 |
| *Standard deviation* | | *8.712* | *7.021* |

Notes: Each column presents the results of an OLS regression analysis. The dependent variables in each column are the number of transplantations from deceased donors per million population plus the number of transplantations from living donors per million population (Total TX), and the number of organ patients who died while on the waiting list per million population (Mortality). All regressions include year fixed effects and dummy variables for missing values on the independent variables. We select countries that did not change their consent system after 1999, and we only include country-year observations for which both the number of transplantations from deceased donors, the number transplantations from living donors, and the number of waiting list deaths are non-missing. Additionally, we only include European countries, Australia, Canada, New Zealand, and the United States (i.e., excluding Latin American, Asian, and African countries). Robust standard errors clustered at the country level are reported in parentheses, *** p<0.01, ** p<0.05, * p<0.1.

Source: Authors’ calculations based on Newsletter Transplant 2002-2016.

**Table O. The relationship between consent systems and the number of transplantations from deceased and living donors (pmp) and the number of patients who died while on the waiting list (pmp), by organ, truncated at the 99^th^ percentile**

|  |  | (1) | (2) |
| --- | --- | --- | --- |
|  |  | Kidney | Liver |
|  |  |  |  |
| Total TX | α_9_: Opt-out (1 = yes) | 4.815** | 5.060*** |
|  |  | (2.309) | (1.347) |
|  | Total deaths all causes (pmp, age-standardized) | 0.011 | -0.106* |
|  |  | (0.133) | (0.063) |
|  | GDP pc (current US$/10,000) | 1.400 | -0.701 |
|  |  | (1.693) | (0.935) |
|  | Health expenditures pc (current US$/1,000) | 4.016*** | 2.794*** |
|  |  | (1.252) | (0.613) |
|  | Religious denomination (% Roman Catholic) | -0.037 | 0.031 |
|  |  | (0.050) | (0.028) |
|  | Constant | 20.496*** | 8.088** |
|  |  | (7.753) | (3.954) |
| Mortality | β_9_: Opt-out (1 = yes) | -0.558 | 0.483 |
|  |  | (1.247) | (0.494) |
|  | Total deaths all causes (pmp, age-standardized) | -0.003 | -0.026 |
|  |  | (0.032) | (0.018) |
|  | GDP pc (current US$/10,000) | -2.301*** | -1.020*** |
|  |  | (0.889) | (0.283) |
|  | Health expenditures pc (current US$/1,000) | 2.314*** | 0.974*** |
|  |  | (0.843) | (0.232) |
|  | Religious denomination (% Roman Catholic) | -0.033 | -0.009 |
|  |  | (0.029) | (0.012) |
|  | Constant | 5.448* | 3.792** |
|  |  | (2.908) | (1.526) |
| Chi2 test: α_9_+ β_9_=0 [*p*-value] | | [0.178] | [0.001] |
|  |  |  |  |
| Observations | | 534 | 485 |
| Number of countries in the estimation sample | | 44 | 39 |
|  |  |  |  |
| Mean total TX rate in estimation sample | | 28.270 | 9.632 |
| *Standard deviation* | | *15.879* | *3.428* |
| Mean organ-patient mortality rate in estimation sample | | 10.617 | 5.441 |
| *Standard deviation* | | *8.712* | *7.482* |

Notes: Each column presents the results of an OLS regression analysis. The dependent variables in each column are the number of transplantations from deceased donors per million population plus the number of transplantations from living donors per million population, truncated at the 99^th^ percentile (Total TX), and the number of organ patients who died while on the waiting list per million population, truncated at the 99^th^ percentile (Mortality). All regressions include year fixed effects and dummy variables for missing values on the independent variables. We select countries that did not change their consent system after 1999, and we only include country-year observations for which both the number of transplantations from deceased donors, the number transplantations from living donors, and the number of waiting list deaths are non-missing. Robust standard errors clustered at the country level are reported in parentheses, *** p<0.01, ** p<0.05, * p<0.1.

Source: Authors’ calculations based on Newsletter Transplant 2002-2016.

**Appendix 4. Main tables including all controls**

**Table P. The relationship between consent systems and the number of transplantations from deceased donors (pmp) and the number of patients who died while on the waiting list (pmp), by organ**

|  |  | (1) | (2) | (3) | (4) |
| --- | --- | --- | --- | --- | --- |
|  |  | Kidney | Liver | Heart | Lung |
|  |  |  |  |  |  |
| DDTX | α_10_: Opt-out (1 = yes) | 7.082*** | 4.357*** | 1.031** | -0.106 |
|  |  | (2.331) | (1.258) | (0.430) | (0.678) |
|  | Total deaths all causes (pmp, age-standardized) | 0.076 | -0.057 | -0.002 | -0.038 |
|  |  | (0.129) | (0.057) | (0.024) | (0.029) |
|  | GDP pc (current US$/10,000) | -3.276 | -0.194 | 0.097 | 0.670 |
|  |  | (2.097) | (0.835) | (0.248) | (0.494) |
|  | Health expenditures pc (current US$/1,000) | 6.763*** | 2.562*** | 0.865*** | 0.359 |
|  |  | (1.910) | (0.551) | (0.180) | (0.344) |
|  | Religious denomination (% Roman Catholic) | 0.054 | 0.049* | 0.013* | 0.003 |
|  |  | (0.039) | (0.027) | (0.007) | (0.010) |
|  | Constant | 8.165 | 3.824 | 1.413 | 1.972 |
|  |  | (7.804) | (3.563) | (1.426) | (2.191) |
| Mortality | β_10_: Opt-out (1 = yes) | -0.811 | 0.434 | -0.171 | -0.331* |
|  |  | (1.423) | (0.494) | (0.280) | (0.197) |
|  | Total deaths all causes (pmp, age-standardized) | 0.010 | -0.020 | 0.002 | -0.007 |
|  |  | (0.031) | (0.018) | (0.011) | (0.007) |
|  | GDP pc (current US$/10,000) | -1.815*** | -0.979*** | -0.036 | 0.252*** |
|  |  | (0.673) | (0.294) | (0.132) | (0.094) |
|  | Health expenditures pc (current US$/1,000) | 1.980** | 0.981*** | 0.034 | -0.116 |
|  |  | (0.776) | (0.234) | (0.116) | (0.110) |
|  | Religious denomination (% Roman Catholic) | -0.028 | -0.008 | -0.004 | 0.000 |
|  |  | (0.028) | (0.013) | (0.006) | (0.004) |
|  | Constant | 4.040 | 3.401** | 1.250* | 1.088* |
|  |  | (2.590) | (1.568) | (0.739) | (0.639) |
| Chi² test: α_10_+β_10_=0 [*p*-value] | | [0.047] | [0.003] | [0.192] | [0.564] |
| Observations | | 549 | 514 | 492 | 390 |
| Number of countries in the estimation sample | | 45 | 42 | 38 | 30 |
| Mean DDTX rate in estimation sample | | 19.849 | 8.510 | 3.121 | 3.134 |
| *Standard deviation* | | *13.056* | *7.295* | *2.575* | *3.208* |
| Mean organ-patient mortality rate in estimation sample | | 4.547 | 1.966 | 0.906 | 0.648 |
| *Standard deviation* | | *5.859* | *1.699* | *0.780* | *0.660* |

Notes: Each column presents the results of a seemingly unrelated OLS regression analysis. The dependent variables in each column are the number of transplantations from deceased donors per million population (DDTX), and the number of organ patients who died while on the waiting list per million population (Mortality). All regressions include year fixed effects and dummy variables for missing values on the independent variables. We select countries that did not change their consent system after 1999, and we only include country-year observations for which both the number of transplantations from deceased donors and the number of deaths while on the waiting lists are non-missing. Robust standard errors clustered at the country level are reported in parentheses, *** p<0.01, ** p<0.05, * p<0.1.

Source: Authors’ calculations based on Newsletter Transplant 2002-2016.

**Table Q. The relationship between consent systems and the number of transplantations from deceased and living donors (pmp) and the number of patients who died while on the waiting list (pmp), by organ**

|  |  | (1) | (2) |
| --- | --- | --- | --- |
|  |  | Kidney | Liver |
|  |  |  |  |
| Total TX | α_11_: Opt-out (1 = yes) | 5.046** | 5.211*** |
|  |  | (2.369) | (1.371) |
|  | Total deaths all causes (pmp, age-standardized) | 0.005 | -0.111* |
|  |  | (0.135) | (0.064) |
|  | GDP pc (current US$/10,000) | 1.596 | -0.740 |
|  |  | (1.846) | (0.957) |
|  | Health expenditures pc (current US$/1,000) | 3.785*** | 2.762*** |
|  |  | (1.445) | (0.638) |
|  | Religious denomination (% Roman Catholic) | -0.044 | 0.026 |
|  |  | (0.053) | (0.029) |
|  | Constant | 20.959*** | 8.516** |
|  |  | (7.931) | (4.070) |
| Mortality | β_11_: Opt-out (1 = yes) | -0.784 | 0.485 |
|  |  | (1.405) | (0.495) |
|  | Total deaths all causes (pmp, age-standardized) | -0.001 | -0.026 |
|  |  | (0.033) | (0.018) |
|  | GDP pc (current US$/10,000) | -2.276** | -1.056*** |
|  |  | (0.899) | (0.288) |
|  | Health expenditures pc (current US$/1,000) | 2.309*** | 1.010*** |
|  |  | (0.840) | (0.239) |
|  | Religious denomination (% Roman Catholic) | -0.031 | -0.009 |
|  |  | (0.029) | (0.012) |
|  | Constant | 5.226* | 3.803** |
|  |  | (3.016) | (1.534) |
| Chi^2^ test: α_11_+β_11_=0 [*p*-value] | | [0.200] | [0.001] |
|  | |  |  |
| Observations | | 534 | 485 |
| Number of countries in the estimation sample | | 44 | 39 |
|  | |  |  |
| Mean total TX rate in estimation sample | | 28.270 | 9.632 |
| *Standard deviation* | | *15.879* | *1.711* |
| Mean organ-patient mortality rate in estimation sample | | 4.618 | 2.026 |
| *Standard deviation* | | *5.906* | *7.482* |

Notes: Each column presents the results of a seemingly unrelated OLS regression analysis. The dependent variables in each column are the number of transplantations from deceased donors per million population plus the number of transplantations from living donors per million population (Total TX), and the number of organ patients who died while on the waiting list per million population (Mortality). All regressions include year fixed effects and dummy variables for missing values on the independent variables. We select countries that did not change their consent system after 1999, and we only include country-year observations for which both the number of transplantations from deceased donors, the number transplantations from living donors, and the number of waiting list deaths are non-missing. Robust standard errors clustered at the country level are reported in parentheses, *** p<0.01, ** p<0.05, * p<0.1.

Source: Authors’ calculations based on Newsletter Transplant 2002-2016.

**Table R. The relationship between consent systems and the number of transplantations from deceased donors (pmp) and the number of patients who died while on the waiting list (pmp) in countries without reports of frequent organ trafficking, by organ**

|  |  | (1) | (2) | (3) | (4) |
| --- | --- | --- | --- | --- | --- |
|  |  | Kidney | Liver | Heart | Lung |
|  |  |  |  |  |  |
| DDTX | α_12_: Opt-out (1 = yes) | 9.333*** | 5.628*** | 1.350** | 0.843 |
|  |  | (2.642) | (1.476) | (0.625) | (0.780) |
|  | Total deaths all causes (pmp, age-standardized) | 0.290*** | -0.069 | 0.001 | 0.002 |
|  |  | (0.097) | (0.084) | (0.029) | (0.029) |
|  | GDP pc (current US$/10,000) | -7.453*** | 1.032 | 0.528 | -0.643 |
|  |  | (1.824) | (1.824) | (0.551) | (0.870) |
|  | Health expenditures pc (current US$/1,000) | 11.704*** | 1.359 | 0.469 | 1.884** |
|  |  | (2.031) | (1.751) | (0.553) | (0.911) |
|  | Religious denomination (% Roman Catholic) | 0.030 | 0.063* | 0.018** | 0.011 |
|  |  | (0.034) | (0.034) | (0.008) | (0.012) |
|  | Constant | -4.276 | 1.705 | 0.446 | -1.454 |
|  |  | (6.207) | (5.063) | (2.029) | (2.478) |
| Mortality | β_12_: Opt-out (1 = yes) | -1.975 | 0.766 | -0.163 | -0.200 |
|  |  | (1.855) | (0.493) | (0.375) | (0.152) |
|  | Total deaths all causes (pmp, age-standardized) | -0.021 | 0.003 | 0.011 | 0.003 |
|  |  | (0.043) | (0.016) | (0.010) | (0.004) |
|  | GDP pc (current US$/10,000) | -0.498 | -0.756 | -0.133 | 0.142 |
|  |  | (0.893) | (0.644) | (0.267) | (0.118) |
|  | Health expenditures pc (current US$/1,000) | 0.469 | 0.922 | 0.211 | 0.062 |
|  |  | (1.033) | (0.644) | (0.269) | (0.113) |
|  | Religious denomination (% Roman Catholic) | -0.003 | 0.011 | 0.006 | 0.005* |
|  |  | (0.026) | (0.008) | (0.004) | (0.002) |
|  | Constant | 4.587 | 0.452 | 0.345 | 0.328 |
|  |  | (3.335) | (1.301) | (0.755) | (0.426) |
| Chi^2^ test: α_12_+β_12_=0 [*p*-value] | | [0.069] | [0.000] | [0.216] | [0.400] |
|  |  |  |  |  |  |
| Observations | | 423 | 398 | 382 | 292 |
| Number of countries in the estimation sample | | 34 | 33 | 29 | 23 |
|  |  |  |  |  |  |
| Mean DDTX rate in estimation sample | | 21.324 | 8.768 | 3.163 | 3.084 |
| *Standard deviation* | | *13.352* | *1.451* | *2.626* | *3.375* |
| Mean organ-patient mortality rate in estimation sample | | 4.444 | 1.762 | 0.893 | 0.602 |
| *Standard deviation* | | *5.875* | *7.652* | *0.765* | *0.579* |

Notes: Each column presents the results of a seemingly unrelated OLS regression analysis. The dependent variables in each column are the number of transplantations from deceased donors per million population (DDTX) and the number of organ patients who died while on the waiting list per million population (Mortality). All regressions include year fixed effects and dummy variables for missing values on the independent variables. We select countries that did not change their consent system after 1999, and we only include country-year observations for which both the number of transplantations from deceased donors and the number of waiting list deaths are non-missing. Additionally, we exclude countries with reports of organ trafficking as either an organ-importing or organ-exporting country (i.e., Australia, Bolivia, Brazil, Canada, Colombia, Egypt, Iran, Israel, Moldova, Peru, Turkey, Ukraine and the United States). Robust standard errors clustered at the country level are reported in parentheses, *** p<0.01, ** p<0.05, * p<0.1.

Source: Authors’ calculations based on Newsletter Transplant 2002-2016.

**Appendix 5. Additional figure**

**Fig 1. The number of deceased-donor transplantations and waiting list deaths per million population, by organ, by year, and by consent system**


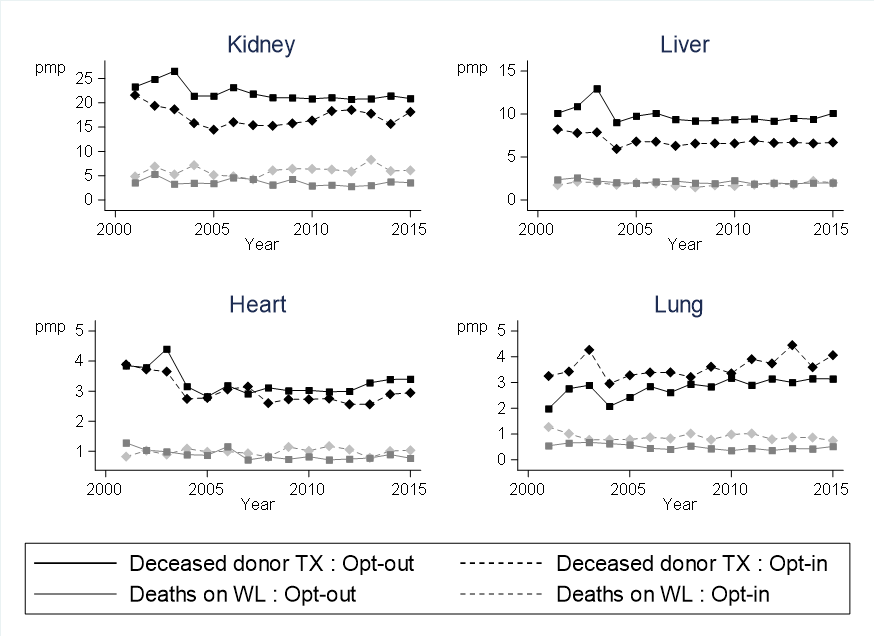


Notes: The graphs are based on the estimation samples for the results shown in Table 2.

Source: Authors’ calculations based on Newsletter Transplant 2002-2016.

**References**

1. Organización Nacional de Trasplantes, *Publicaciones.* Retrieved from http://www.ont.es/publicaciones/Paginas/Publicaciones.aspx on Dec 9, 2016.
2. R. Bouwman, J. Lie, M. Bomhoff, R.D. Friele. *The ACTOR-study: Study on the set-up of organ donation and transplantation in the EU Member States, uptake and impact of the EU Action Plan on Organ Donation and Transplantation (2009-2015)*, Utrecht: NIVEL (2013).
3. A.M. Rosenblum, L.D. Horvat, L.A. Siminoff, V. Prakash, J. Beitel, A.M. Garg. The authority of next-of-kin in explicit and presumed consent systems for deceased organ donation: an analysis of 54 nations. *Nephrology Dialysis Transplantation* **0**, 1-14 (2011).
4. L. Shepherd, R.E. O'Carroll, E. Ferguson. An international comparison of deceased and living organ donation/transplant rates in opt-in and opt-out systems: a panel study. *BMC Medicine* **12** (131) (2014).
5. K. Habchi, A. Benziane, K. Toumi, B. Mohamed. Panorama of Transplantation in Algeria, In: ELPAT (ed), *4th ELPAT Congress: Ethical, Legal and Psychosocial Aspects of Transplantation, Global Challenges, Programme & Abstract Book*, 96 (2016).
6. G. Balbinotto Neto, E. Nunes da Silva, A.K. Campelo. The impact of presumed consent law on organ donation: an empirical analysis from quantile regression for longitudinal data. *Working Paper of Public Health* **3**, 1-22 (2012).
7. G. Balbinotto Neto, E. Nunes da Silva, A.K. Campelo. The impact of presumed consent law on organ donation: an empirical analysis from quantile regression for longitudinal data. *Berkely Program in Law & Economics* (2007).
8. R. Mizraji, I. Alvarez, R.I. Palacios, C. Fajardo, C. Berrios, F. Morales, E. Luna, C. Milanés, M. Andrade, E. Duque, F. Giron, J. Alfonso, S. Herra, C. Soratti, R. Ibar, V.D. Garcia. Organ donation in Latin America. *Transplantation Proceedings* **39** (2), 333–335 (2007).
9. D. Austen, E. Skewes. Organ Donation and Transplantation, In: D. Elliott, L. Aitken, W. Chaboyer (eds.) *ACCCN's Critical Care Nursing*, 746-762 (2012).
10. C. Bigg. 'Gutted like a pig': grieving mother takes on Russia's organ donation system, Eurasianet.org. Retrieved from http://www.eurasianet.org/node/77806 on Dec 19, 2016.
11. P. Flores. Culture hinders organ donations, transplants in Bolivia, Honolulu Star Advertiser (2015). Retrieved from http://www.staradvertiser.com/2015/02/21/breaking-news/culture-hinders-organ-donations-transplants-in-bolivia/ on 19 Dec 2016.
12. G.O. Phillips. *Legal Basis of Global Tissue Banking: A Proactive Clinical Perspective,* World Scientific (2015).
13. N. Hicko. Croation model the possible answer to lack of donors in Balkan countries? Balkan Science Report (2013). Retrieved from http://www.balkansciencereport.com/2013/09/croatian-model-possible-answer-to-lack.html. on Dec 19, 2016.
14. G. Kekenadze, I. Javakhishvili, D. Kekenadze, S. Rustaveli. Legal and Ethical Aspects of Organ Transplantology in Georgia. Global international scientific analytical project (2011). Retrieved from http://gisap.eu/node/1028#. on Dec 19, 2016.
15. R. Garcia-Gallont, R. Matesanz, F. L. Delmonico. Organ donation and transplantation in Central America. *Transplantation* **99** (3), 459-460 (2015).
16. Bundeszentrale für gesundheitliche Aufklärung. *The Law on Organ Donation in German: A Declaration-based Solution.* Retrieved from https://www.organspende-info.de/sites/all/files/files/BZGA-15-02525_Flyer_Entscheidungslosung_EN.pdf on Dec 19, 2016.
17. Global Observatory on Donation and Transplantation. Region: The Americas, Country: Dominican Republic. Retrieved from http://ont.es/americas/Organizational%20and%20legal%20aspects/Dominican%20Republic%20profile.pdf. on Dec 19, 2016.
18. S.M. Kazemeyni, A.R. Bagheri, A.R. Heidary. Worldwide cadaveric organ donation systems (transplant organ procurement). *Urology Journal* **1** (3), 157-164 (2004).
19. M. Tumin, K. Tafran, R. Hazli Zakaria, N.H. Mohd Satar, K. Peng Ng, S. Kun Lim. Do family members of dialysis patients have a positive attitude toward organ donation? *Annals of Transplantation* **20**, 752-756 (2015).
20. Parliamentary Secretariat for Health. *Proposal for Legislation on Organ and Tissue Donation: A Maltese Government White Paper* (2015) Retrieved from https://socialdialogue.gov.mt/en/Public_Consultations/MEH-HEALTH/Documents/L-05-2015%20-%20White%20Paper%20on%20Organ%20and%20Tissue%20donation/Organ%20Donation-White%20paper%20for%20consultation%20(2).pdf on Dec 19, 2016.
21. European Court of Human Rights. Case of Petrova v. Latvia. Application no. 4605/05 (2014).
22. E. Kelly. International organ trafficking crisis: solutions addressing the heart of the matter. *Boston College Law Review* **54** (3), 1317-1349 (2013).
23. UNESCO Cairo Office. Ethics and Law in Biomedicine and Genetics: An Overview of National Regulations in the Arab States. Cairo, Egypt: United Nations Education, Scientific and Cultural Organization (2011).
24. S.G. Stetsenko, V.Y. Stetsenko, I. C. Senyuta. *Medical law Ukraine* (2008)
25. Republica Oriental del Uruguay, *Donación y Transplante de Células, Órganos y Tejidos* (Ley Nº 18.968, 2012) Retrieved from http://200.40.229.134/Leyes/AccesoTextoLey.asp?Ley=18968&Anchor= on Dec 19, 2016.
26. Socialist Republic of Vietnam. Law on donation, removal and transplantation of human tissues and organs and donation and recovery of cadavers (2006).
27. Statistics Eurotransplant. Retrieved from <http://statistics.eurotransplant.org/index.php?search_type=WL+removals&search_organ=by+organ&search_region=by+country&search_period=2007&search_characteristic=removal+reason&search_text=9023> on Oct 23, 2017.
28. Statistics Scandiatransplant. Retrieved from [http://www.scandiatransplant.org/data.](http://www.scandiatransplant.org/data.%20) on Oct 23, 2017.
29. Statistics from the Agence Biomedicine. Retrieved from: <https://www.agence-biomedecine.fr/Procurement-and-transplantation-of,530?lang=fr> on Oct 23, 2017.
30. Statistics on UK organ donation. Retrieved from: [https://nhsbtdbe.blob.core.windows.net/umbraco-assets-corp/4496/section_5_kidney_activity.pdf](https://nhsbtdbe.blob.core.windows.net/umbraco-assets-corp/4496/section_5_kidney_activity.pdf%20on%2023%20Oct%202017) on Oct 23, 2017.
31. Statistics from the OPTN. Retrieved from: [https://optn.transplant.hrsa.gov/data/view-data-reports/national-data/#](https://optn.transplant.hrsa.gov/data/view-data-reports/national-data/) on Oct 23, 2017.
32. Statistics on organ donation in New Zealand and Australia. Retrieved from: [http://www.anzdata.org.au/anzod/ANZODReport/2016/2016-anzod-12-waitinglist_v2.0_20170114.pdf](http://www.anzdata.org.au/anzod/ANZODReport/2016/2016-anzod-12-waitinglist_v2.0_20170114.pdf%20on%2023%20Oct%202017) on Oct 23, 2017.
33. A. Rithalia, C. McDaid, S. Suekarran, L. Myers, A. Sowden. Impact of presumed consent for organ donation on donation rates: a systematic review. *BMJ* **338**, 1-8 (2009).
34. A. Abadie, S. Gay. The impact of presumed consent legislation on cadaveric organ donation: a cross-country study. *Journal of Health Economics* **25** (4), 599-620 (2006).
35. R. Coppen, R. Friele, R. Marquet, S. Gevers, S. Opting-out systems: no guarantee for higher donation rates. *Transplant International* **18**, 1275-1279 (2005).
36. R. Gimbel, M. Strosberg, S. Lehrman, E. Gefenas, F. Taft. Presumed consent and other predictors of cadaveric organ donation in Europe. *Progress in Transplantation* **13** (1), 17-23 (2003).
37. K. Charpentier, A. Mavanur. Removing patients from the liver transplant waiting list: a survey of US liver transplant programs. *Liver Transplantation* **14**, 303-307 (2008).
38. Shimazono, Y. (2007). The state of the international organ trade: a provisional picture based on integration of available information. Bulletin of the World Health Organization 85 (12), 901-980.

1. To avoid confusion, please note that there is a difference between transplantations and donors; a donor is the person from whom at least one organ is obtained in order to transplant that organ into an organ patient. There is no use in becoming an organ donor (living or deceased) if there is no potential recipient (who is a good match and has a high probability of survival). This means that by construction, each donor equals at least one, but potentially multiple, transplantation(s). [↑](#footnote-ref-1)
2. The data are available in pdf documents (<http://www.ont.es/publicaciones/Paginas/Publicaciones.aspx>). [↑](#footnote-ref-2)
3. <https://data.worldbank.org/indicator/NY.GDP.PCAP.CD> (retrieved on June 1, 2018). [↑](#footnote-ref-3)
4. <http://apps.who.int/nha/database/Select/Indicators/en> (retrieved on June 1, 2018). For all countries, this measure of health expenditures includes all health care goods and services used or consumed during a year, i.e. the sum of the domestic general government health expenditures, the domestic private health expenditures, and the health expenditures from external sources. External sources comprise direct foreign transfers and foreign transfers distributed by governments encompassing all financial inflows into the health system from outside the country. [↑](#footnote-ref-4)
5. <http://apps.who.int/healthinfo/statistics/mortality/whodpms/> (retrieved on June 1, 2018). [↑](#footnote-ref-5)
6. In 2006 the Human Tissue Act was introduced in Scotland, which made it illegal to remove or store human tissue without consent. [↑](#footnote-ref-6)
7. Already excluded from all analyses due to a lack of (reliable) information on the national consent system for organ donation. [↑](#footnote-ref-7)
